# Phase separation and ageing of glycine-rich protein from tick adhesive

**DOI:** 10.1038/s41557-024-01686-8

**Published:** 2024-11-29

**Authors:** Ketan A. Ganar, Manali Nandy, Polina Turbina, Chang Chen, Dennis Suylen, Elisa Nihoul, Emily Louise Pascoe, Stan van der Beelen, Maarten Plaum, Leendert van den Bos, Constantianus J. M. Koenraadt, Ingrid Dijkgraaf, Siddharth Deshpande

**Affiliations:** 1 Laboratory of Physical Chemistry and Soft Matter, Wageningen University and Research, Wageningen, the Netherlands; 2 Department of Biochemistry, Cardiovascular Research Institute Maastricht (CARIM), Maastricht University, Maastricht, the Netherlands; 3 Laboratory of Entomology, Wageningen University and Research, Wageningen, the Netherlands; 4 EnzyTag BV, Nuth, the Netherlands; 5 Present Address: Conservation Genomics Research Unit, Research and Innovation Centre, Fondazione Edmund Mach, San Michele All’Adige, Trento, Italy

**Keywords:** Intrinsically disordered proteins, Biophysical chemistry, Peptides

## Abstract

Hard ticks feed on their host for multiple days. To ensure firm attachment, they secrete a protein-rich saliva that eventually forms a solid cement cone. The underlying mechanism of this liquid-to-solid transition is currently not understood. This study focuses on the phase transitions of a disordered glycine-rich protein (GRP) found in tick saliva. We show that GRP undergoes liquid–liquid phase separation via simple coacervation to form biomolecular condensates in salty environments. Cation–*π* and *π*–*π* interactions mediated by periodically placed arginine and aromatic amino-acid residues are the primary driving forces that promote phase separation. Interestingly, GRP condensates exhibit ageing by undergoing liquid-to-gel transition over time and exhibit adhesive properties, similar to the naturally occurring cement cone. Finally, we provide evidence for protein-rich condensates in natural tick saliva. Our findings provide a starting point to gain further insights into the bioadhesion of ticks, to develop novel tick control strategies, and towards achieving biomedical applications such as tissue sealants.

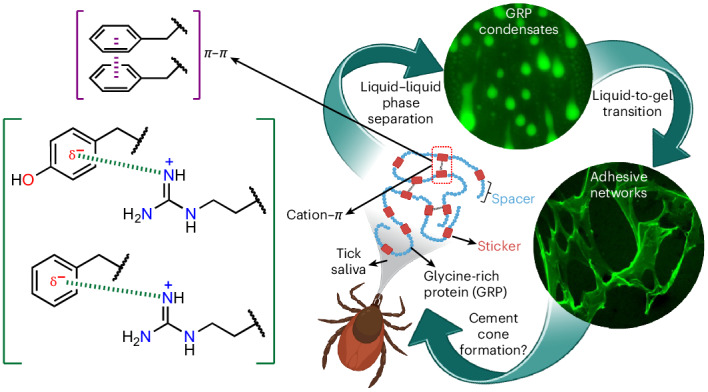

## Abstract (web summary)

Ticks adhere firmly to their host by secreting a protein-rich saliva that eventually forms a solid bioadhesive, but the mechanism underlying this phase transition is not understood. Now it has been shown that a disordered glycine-rich protein present in tick saliva undergoes liquid–liquid phase separation, followed by a liquid-to-gel transition, and exhibits adhesive properties.

## Main

Biological adhesives are sticky materials used by a wide variety of organisms for different purposes, including attachment, prey capture, locomotion, building and defence^1^. The range of species that can produce bioadhesives is diverse, from bacterial biofilms and slimy slugs, to stickleback nests and sticky spiderwebs^2^. Many animals use protein-based adhesives. For example, sandcastle worms build reef-like mounds^3^, mussels attach via proteinaceous threads^4^, and velvet worms eject slime to entangle their prey^5^. Although some of these bioadhesives have been studied in detail, knowledge regarding the adhesive mechanisms of many others is largely lacking. One unexplored and unique bioadhesive is produced by ticks, a widespread parasite of public-health and economic importance^6,7^.

**Fig. 1:**
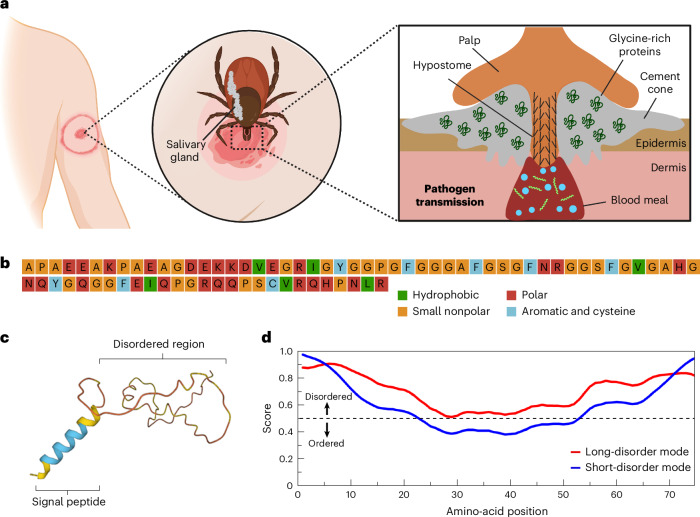
Glycine-rich protein present in tick saliva is intrinsically disordered and shows a high propensity for LLPS. **a**, Schematic overview showing the consequence of a tick bite. The tick inserts its hypostome into the host epidermis and secretes a protein-rich saliva, abundant in GRPs. The saliva undergoes a liquid-to-solid transition, forming a hard cement cone, allowing the tick to feed on the host over several days and facilitating pathogen transmission (the shown ‘bull’s-eye’ rash is typical in the case of *Borrelia* infection, causing Lyme borreliosis). **b**, Amino-acid composition of tick-GRP77 shows a high proportion of non-polar amino acids (44%, of which 26% are glycine). **c**, AlphaFold correctly predicts the N-terminal signal peptide of GRP as an α-helix, while the rest of the sequence (tick-GRP77) remains unstructured, indicating a disordered region. **d**, The IUPred2A long disorder mode scores of the entire tick-GRP77 sequence are above 0.5, while the short disorder score shows prominent disorder near the termini, overall indicating tick-GRP77 to be a highly disordered protein.

Ticks are arthropods that feed on their host by sucking blood over a prolonged period, usually multiple days in the case of hard ticks^8^. Importantly, the prolonged contact and transfer of tick saliva to the host can lead to pathogen transmission and consequently disease, notable examples being Lyme borreliosis in humans, and babesiosis, anaplasmosis and heartwater in bovine species^9,10^. To feed successfully, hard ticks attach to the host in two stages: an initial mechanical attachment, followed by bioadhesive production to create what is known as the cement cone^11^ (Fig. 1a). Mechanical attachment is achieved using mouthparts consisting of two palps that perform sensory functions, a pair of chelicerae to cut into the host tissue, and a hypostome that acts as a channel for blood as well as to penetrate the outermost layer of the epidermis^11,12^ (Supplementary Fig. 1). Upon attachment, saliva is secreted from the salivary glands. This milky-white proteinaceous fluid has adhesive properties and undergoes a liquid-to-solid transition once exposed to air, therefore bearing the name ‘cement‘^11,13,14^. A cement cone, resembling a wedge-shaped anchor, is formed around the incision site of the tick, which strengthens attachment to the host^11^. Feeding begins thereafter, with the formation of small blood pools under the skin, followed by intermittent ejection of saliva^15^. However, the exact mechanism and identity of the key salivary components responsible for the formation of the cement cone remain unknown.

The cement cone is protein-rich, containing tick peptides and proteins, host proteins, as well as non-peptidic molecules, exosomes and so on^11,15,16^. Biochemical and bioinformatic analyses have revealed that glycine-rich proteins (GRPs) are abundant in tick saliva^17–20^. For example, a recent study found that 19% of the identified protein sequences from the cement cone belonged to GRPs, and the expression of GRPs increased substantially during blood feeding^21^. GRPs have been associated with providing strength, insolubility and stabilization to the cement cone^11^, but the mechanism by which GRPs facilitate these functions remains unknown. Glycine-rich regions are known to be commonly present in intrinsically disordered proteins (IDPs) because they can prevent protein folding due to their small size and high degree of freedom^22^. Liquid–liquid phase separation (LLPS), manifested through coacervation, is driven by the interactions between multivalent biomolecules such as proteins and nucleic acids, resulting in polymer-rich condensate droplets that are in equilibrium with a polymer-depleted phase^23^. IDPs have often been associated with LLPS because of their capacity to undergo large-scale conformational changes and establish multiple interactions with neighbouring molecules^24,25^. Condensates have been found to play crucial roles in diverse cellular functions^26–29^, and although usually liquid-like, they have been shown to undergo a liquid-to-solid transition^5,30–33^. LLPS has indeed been shown to play a role in bioadhesion via liquid-to-solid transition. Various aquatic and terrestrial organisms, such as mussels, sandcastle worms, spiders and velvet worms, have been shown to utilize LLPS to produce strong adhesives under specific environmental triggers such as crosslinking, pH changes and evaporation^3,5,32,34–38^.

This strong link between IDPs, LLPS and liquid-to-solid transition prompted us to explore the possible role of tick GRPs in cement cone formation. In this article, we study a tick GRP from the hard tick species *Ixodes scapularis* and systematically investigate its evaporation- and salt-induced LLPS, the underlying molecular interactions, and the capacity of condensates to age into solid-like aggregates as well as to exhibit adhesive properties. Our findings aim to shed light on cement cone formation and the possible role of salivary GRPs by providing the necessary solidification and adhesive properties. The obtained knowledge may become relevant for tick prophylaxis and for biotechnological applications, such as medical sealants.

## Results

### Sequence analysis suggests GRP is a disordered protein

We selected one of the identified GRPs present in tick (*I. scapularis*) saliva (Fig. 1), simply known as GRP (UniProt Q4PME3) and we refer to it as such hereon. The first 19 N-terminal amino acids of GRP (sequence, ^1^MNRMFVLAATLALVGMVFA^19^) constitute the signal peptide necessary for its translocation. The remaining 77 amino acids constitute the mature GRP sequence (20–96) and we refer to it as tick-GRP77 hereon (note that the amino-acid locations within tick-GRP77 are renumbered, that is, the first position refers to the 20th amino acid and the 77th is the 96th one). As the name suggests, tick-GRP77 is rich in glycine residues (~26%) with most of them located in the middle of the sequence. Sequence composition analysis by PSIPRED^39^ revealed a large fraction of other non-polar (~44%; mainly alanine and proline) and polar (~36%; mainly glutamic acid, arginine and glutamine) amino acids. In comparison, the fraction of hydrophobic (~7%; valine and isoleucine) and aromatic (~9%; tyrosine and phenylalanine) residues was relatively small (Fig. 1b). Computationally predicting the GRP structure with AlphaFold^40^ showed two distinct regions (Fig. 1c). The signal peptide showed an α-helical conformation, as expected. However, the remaining sequence gave a very low confidence score, hinting at a lack of any kind of secondary structure. The IUPred algorithm^41^, which predicts IDRs, also determined the entire tick-GRP77 to be highly disordered, with a score above 0.5 for the majority of the sequence (Fig. 1d). Multiple other algorithms also predicted the tick-GRP77 amino-acid sequence to be primarily disordered (Supplementary Fig. 2). We further evaluated the sequence using CIDER^42^, which plots the fraction of negatively charged versus positively charged residues. The tick-GRP77 sequence was observed to fall in the same region as many well-characterized phase-separating IDR-containing proteins like FUS^43^ and viral nucleocapsid^44^ (Supplementary Fig. 3). Cumulatively, our bioinformatic analysis indicated a strong inclination for tick-GRP77 to undergo LLPS.

### Tick-GRP77 undergoes LLPS via simple coacervation

**Fig. 2:**
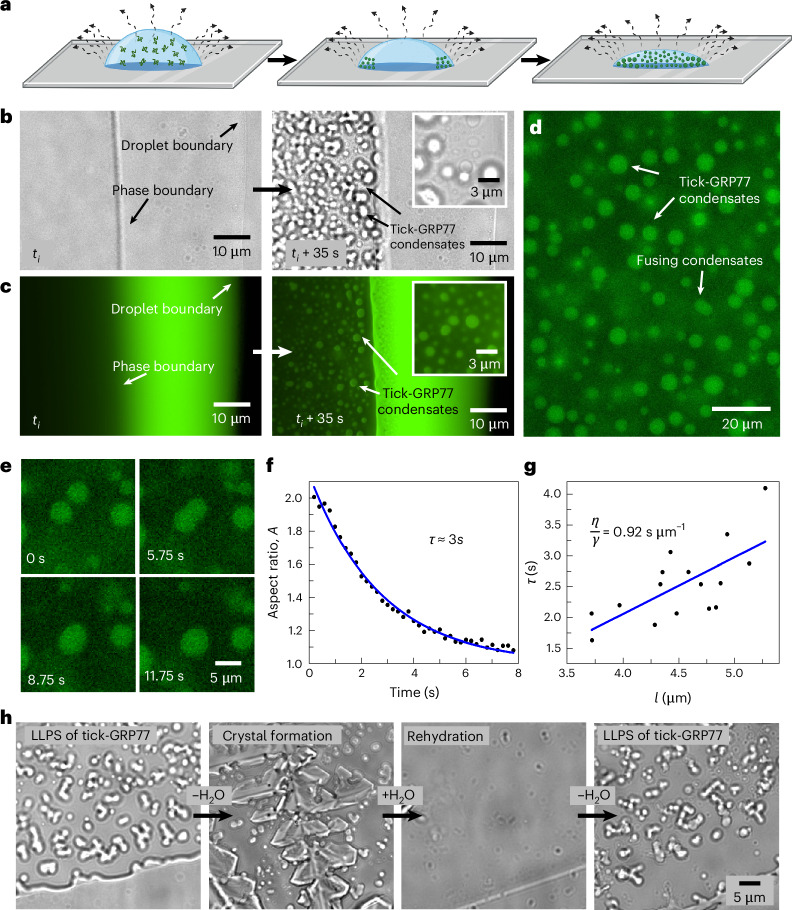
Tick-GRP77 undergoes LLPS via simple coacervation to form liquid-like condensates. **a**, Schematic of the droplet evaporation assay, where a droplet of a buffered tick-GRP77 solution is allowed to evaporate at room temperature, continuously increasing the protein concentration at the droplet boundary. **b**,**c**, Evaporation of a 2-µl tick-GRP77 droplet leads to the formation of spherical condensates near the phase boundary (*t*_i_ ≈ 15 min). Subsequently, condensate formation rapidly spreads inward. Bright-field images are shown in **b**, with corresponding fluorescence images shown in **c**. **d**, Evaporation of tick-GRP77 on a surface-passivated glass slide showing numerous spherical tick-GRP77 condensates. **e**, Two similar-sized condensates coalescing and relaxing into a bigger condensate over a time span of a few seconds. **f**, For a typical example, change in aspect ratio as a function of time during a fusion event. The line shows an exponential decay fit (*R*^*2*^ = 0.99), giving a fusion timescale of *τ* ≈ 3 s. **g**, A linear fit for the decay time (*τ*) against the characteristic length scale (*l*) for several fusion events (*n* = 16) gives an inverse capillary velocity (*η*/*γ*) of 0.92 s µm^−1^ (*R*^*2*^ = 0.47). **h**, The phase-separated solution eventually dries out, forming salt crystals. However, rehydration of the dried sample resolubilizes the condensates. Evaporation of the resolubilized sample again leads to LLPS, demonstrating the reversibility of the phase separation process. The starting concentration of tick-GRP77 was 32 µM in PBS for all the experiments. In the case of fluorescence imaging, the samples were doped with 5 mol% OG488-GRP77. Source data

Following confirmation of the disordered nature of tick-GRP77 via structure prediction analysis, we proceeded with gathering experimental evidence for its LLPS behaviour. We synthesized tick-GRP77 via solid-phase peptide synthesis (SPPS) and native chemical ligation (NCL) (see Methods for details). Because the secretion of tick saliva locally enriches its components in the host tissue, aided by water loss through evaporation, we mimicked this via a straightforward droplet evaporation assay (Fig. 2a). We deposited a small sessile droplet (2 µl) of buffered tick-GRP77 solution (16–500 µM in phosphate-buffered saline (PBS), pH 7.4) on a hydrophilic glass slide at ambient temperature. Over the course of several minutes, evaporation-driven internal flow led to the deposition and up-concentration of tick-GRP77 molecules at the droplet boundary^45^. For better visualization, we added 5 mol% fluorescently labelled tick GRP, OG488-GRP77 (fluorescent label, Oregon Green 488; details are provided in Methods) to the sample. Figure 2b,c presents a time-lapse of an evaporating droplet boundary of a 32 µM tick-GRP77 solution (also Supplementary Video 1).

Fluorescence imaging revealed an intense fluorescence at the droplet boundary over time, indicating an increasing local concentration of the protein (Fig. 2c). Interestingly, this was followed by a sudden appearance of a phase boundary, forming a rim at the droplet boundary. We defined *t*_i_ as the time interval between droplet deposition and formation of this rim. Within a minute after the rim was established (*t*_i_ ≈ 15 min), we observed the sudden appearance of numerous micrometre-sized droplets near the rim (Fig. 2b, right). The GRP-rich nature of these droplets was evident from the fluorescence images (Fig. 2c, right). Repeating the evaporation assay on a surface-passivated glass slide with 5% wt/vol polyvinyl alcohol (PVA; details are provided in Methods) gave a clearer picture of the micrometre-sized, spherical tick-GRP77 condensates (Fig. 2d).

The rim itself represents a condensate phase, where the continuous phase is the protein-rich condensed phase, interspersed with dilute phase droplets, and forms a sharp phase boundary with the rest of the droplet. This rim formation probably happens because of the rapid accumulation of proteins at the boundary, and has been observed for other evaporating condensate-forming protein samples^46^. Further confirmation of this dense continuous phase comes from the fact that we observed fusion of tick-GRP77 condensates with the rim, and a closer look also revealed GRP-depleted dilute phase droplets (Supplementary Fig. 4), similar to earlier reports^46^.

As coacervation is a concentration-dependent process, one would expect the time until the onset of coacervation to be dependent on the initial protein concentration. Indeed, increasing the initial protein concentration from 16 to 128 µM gradually decreased the time required for the onset of coacervation, from 12.7 ± 0.7 min (mean ± s.d.) to 7.5 ± 0.9 min (Supplementary Fig. 5). To further validate the tick-GRP77-specific nature of the condensates, we performed several negative controls (Supplementary Fig. 6): evaporation of tick-GRP77 solubilized in 140 mM NaCl led to similar condensate formation, suggesting that salt-bridging between cationic amino acids and phosphates is not the key driving force for phase separation. However, tick-GRP77 solubilized in Milli-Q water did not form condensates, suggesting a clear role of salts in promoting phase separation, probably via charge screening which allows favourable intermolecular interactions to take place. The use of bovine serum albumin (a globular protein without disordered regions) did not lead to LLPS. Similarly, evaporation of a protein-free buffer solution did not result in any kind of LLPS behaviour.

One of the hallmark properties of condensates is their coalescence behaviour due to surface energy minimization and rapid reorganization within the liquid droplet^26,47^. We readily observed numerous fusion events between tick-GRP77 condensates upon physical contact with each other, followed by their relaxation into a bigger spherical condensate (Fig. 2e and Supplementary Video 2). Tracking the aspect ratio (major to minor axis ratio) of the fusing droplets showed an exponential relaxation to a sphere^48,49^ (Fig. 2f shows a typical example). Following the relation *τ* ≈ *l*(*η*/*γ*), where *l* is the average radius of the fusing droplets, *η* is the viscosity of the droplet, and *γ* is the surface tension, a plot of *τ* against *l* gave us an inverse capillary velocity (*η*/*γ*) of 0.92 s µm^−1^ (Fig. 2g; *n* = 16; *R*^2^ = 0.47; see Methods for details), implying that micrometre-sized tick-GRP77 condensates behaved like a liquid on a timescale longer than a second^49^.

Another key property of LLPS is its reversible nature, at least over short timescales. We checked this by repeating the evaporation assay (32 µM GRP in PBS) to form condensates and immediately rehydrating the sample. As expected, we observed the formation of tick-GRP77 condensates and, after complete evaporation, the sample crystallized due to the presence of salts (Fig. 2h). However, on rehydrating the crystallized sample with 2 µl of Milli-Q water, we immediately observed complete resolubilization of the condensates. This clearly demonstrated the reversible nature of the LLPS process. Following the second evaporation cycle, GRP condensates reformed in a similar manner. In conclusion, tick-GRP77 was observed to undergo simple coacervation and form viscous liquid droplets.

### LLPS is driven primarily by arginine and aromatic residues

**Fig. 3:**
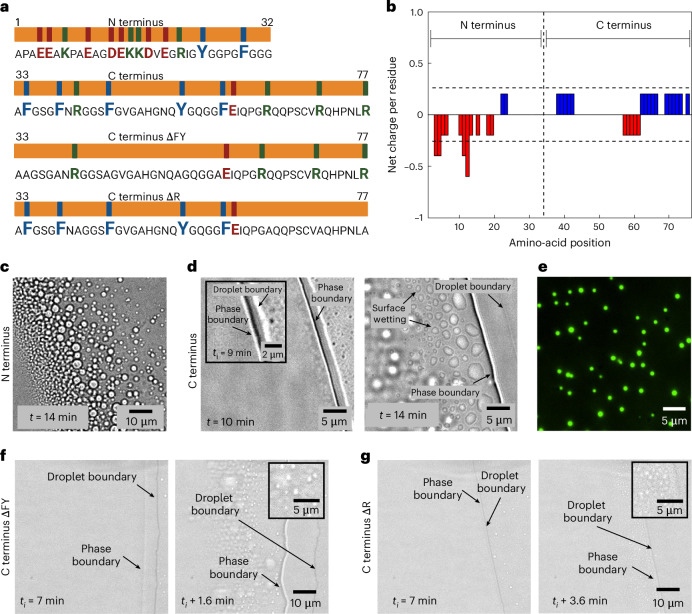
Arginine and aromatic residues are the key drivers of phase separation of tick-GRP77. **a**, The four fractions of tick-GRP77: N terminus (1–32), C terminus (33–77), C terminus mutant without aromatic amino acids (ΔFY mutant) and C terminus mutant without arginine residues (ΔR mutant). They all have a similar glycine content but varying basic (green), acidic (red) and aromatic (blue) amino-acid residues. **b**, Net charge per residue as a function of amino-acid position for the two termini, obtained using CIDER^42^. The N terminus is strongly negatively charged, and the C terminus is moderately positively charged. **c**, Droplet evaporation assay of the N terminus leads to phase separation and the formation of micrometre-sized condensates on a similar timescale as that of tick-GRP77 (*t*_i_ ≈ 13 min). **d**, Droplet evaporation assay of the C terminus leads to quicker phase separation (*t*_i_ ≈ 9 min), shown in the inset. The formed condensates readily wet the glass surface. **e**, Performing the evaporation assay on a PVA-passivated glass slide prevented surface wetting, leading to the formation of spherical C terminus condensates. OG488-GRP77 (5 mol%) readily partitioned in the condensates. **f**, Droplet evaporation assay of the ΔFY mutant led to weak phase separation, with *t*_i_ ≈ 7 min. **g**, Droplet evaporation assay of the ΔR mutant led to negligible phase separation, with *t*_i_ ≈ 7 min. The starting peptide concentrations were 50 µM for all panels. All experiments were performed in PBS.

To identify the protein regions responsible for phase separation and the underlying intermolecular interactions, we experimented with two distinct fractions of tick-GRP77: a 32-amino-acid-long N terminus (20–51) and the remaining 45-amino-acid-long C terminus (52–96), as depicted in Fig. 3a. Both fractions are predicted to be disordered (Fig. 1d) and have comparable glycine content, 9 and 11 residues, respectively. One clear difference is that the N terminus is rich in acidic amino acids, giving it a net negative charge of −3.4 at pH 7.4. By contrast, the C terminus is relatively rich in basic amino-acid residues, giving it a net positive charge of 2.5 at pH 7.4 (Fig. 3b). Conducting droplet evaporation assays for both fractions under identical conditions (50 µM solutions in PBS, pH 7.4) revealed a significant difference between the onset time of coacervation for the two termini. The N terminus showed a similar timescale as tick-GRP77, with *t*_i_ ≈ 13 min (condensates at *t* = 14 min are shown in Fig. 3c). On the other hand, the C terminus underwent phase separation much more quickly (*t*_i_ ≈ 9 min) and also showed a strong tendency to wet the glass surface (Fig. 3d). Passivating the surface with 5% wt/vol PVA led to the formation of spherical condensates, with OG488-GRP77 (5 mol%) readily partitioning within them (Fig. 3e). Despite having equal numbers of cationic amino acids, the tendency of these two fractions to coacervate is clearly different under same salt concentrations, indicating that salt-bridging is not a dominant mechanism for coacervation.

Apart from electrostatic interactions, *π*–*π*, cation–*π*, hydrophobic and hydrogen bonding also play important roles in the formation of condensates^24^. Figure 3a shows that the N terminus contains four cationic (three lysine (K) and one arginine (R)) and two aromatic (one tyrosine (Y) and one phenylalanine (F)) residues. The C terminus, on the other hand, contains four cationic (all R) and five aromatic (four F and one Y) residues, and thus can form more extensive cation–*π* interactions compared with the N terminus. Furthermore, the aromatic amino acids in the C terminus are interspersed and separated by non-aromatic units that can be compared with the spacer-and-sticker model and are capable of forming *π*–*π* interactions^50,51^. Previous reports also suggest that simple coacervation is more favourable if the protein contains relatively hydrophobic stickers and polar spacers, which seems to be the case for the C terminus with multiple serine, asparagine and glutamine residues^52^. Finally, arginine–glycine domains have been reported to form cation–*π* interactions with phenylalanine and thus promote LLPS^53,54^. To test whether aromatic amino acids (F and Y) play an active role in driving phase separation, we synthesized a mutant variant of the C terminus by replacing F and Y (ΔFY mutant) with a non-polar amino-acid alanine (A), keeping the length of the terminus the same as that of the wild type. Evaporation of a 2-µl sessile droplet of ΔFY mutant (50 µM in PBS) showed weak phase separation (*t*_i_ ≈ 7 min, Fig. 3f). Increasing the initial concentration to 100 µM led to normal phase separation (Supplementary Fig. 7). This suggests that the aromatic amino acids capable of forming *π*–*π* interactions are crucial to induce phase separation of the C terminus, but are nonetheless not strictly necessary at high protein concentrations.

On the other hand, arginine (prominent in the C terminus) has been proven to be more hydrophobic than lysine (prominent in the N terminus) due to the presence of a *π*-electron-rich guanidium group, which allows them to form *π*–*π* bonds along with cation–*π* bonds^55^. To test the importance of arginine in promoting phase separation, we replaced the R residues in the C terminus with A (ΔR mutant). Evaporation of a 2-µl sessile droplet of 50 µM ΔR mutant showed almost no phase separation (*t*_i_ ≈ 7 min, Fig. 3g). Even after increasing the initial concentration to 100 µM, the formation of condensates was negligible (Supplementary Fig. 7), indicating that R plays an even more important role in driving phase separation. Comparing the two mutants, we conclude that although both aromatic (F and Y) and arginine (R) residues are needed for optimum phase separation, arginine-based cation–*π* and *π*–*π* interactions play a dominant role in driving the LLPS of tick-GRP77.

We further carried out chemical disruption experiments to probe the role of hydrogen bonding and hydrophobic interactions in the coacervation process (details are provided in Methods). We tested the role of hydrogen bonding using urea, which efficiently forms hydrogen bonds with the amide moieties^56^. Microscopic visualization showed both N and C terminus condensates immediately dissolved in the presence of ~0.5 M urea (Supplementary Fig. 8), indicating an active participation of hydrogen bonding between the peptide backbone of GRP as well as amino-acid residues such as histidine and tyrosine. To check the role of hydrophobic forces in driving LLPS, we used 1,6-hexanediol (1,6-HD), which is widely used to dissolve liquid condensates, by disrupting hydrophobic protein–protein interactions. We observed that N terminus condensates dissolved already in the presence of ~15 mM 1,6-HD, whereas C terminus condensates remained unaffected until ~70 mM 1,6-HD and dissolved completely only at ~140 mM (Supplementary Fig. 8). These results clearly show that hydrophobic interactions also actively participate in LLPS, in particular at the C terminus.

Based on the investigations of the N and C termini, mutant studies, chemical disruption and previous studies^50–52,55,57,58^, we conclude that both cation–*π* and *π*–*π* interactions, particularly those involving arginine residues alongside aromatic ones, are important for the phase transitioning of tick-GRP77. The observed delay in the initiation of coacervation for the N terminus compared with the wild type C terminus can be attributed to the lack of these interactions as well as the electrostatic repulsion between negatively charged amino-acid residues. One can thus regard the C terminus of tick-GRP77 as the key promoter of LLPS, while the N terminus acts as a regulator of the LLPS process.

### Tick-GRP77 forms condensates in the presence of phosphate salts

**Fig. 4:**
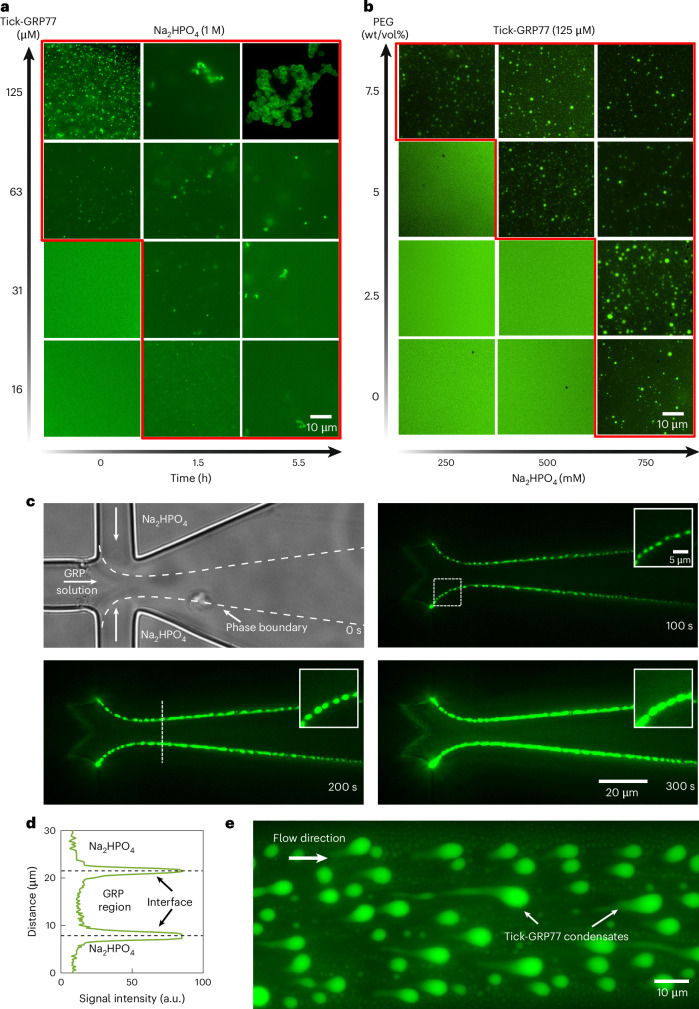
Tick-GRP77 forms condensates in the presence of phosphate salts. **a**, Phase diagram showing the LLPS behaviour of tick-GRP77 in the presence of 1 M Na_2_HPO_4_ as a function of protein concentration and incubation time. **b**, Phase diagram demonstrating that the addition of crowding agents (PEG, 8 kDa) drastically lowers the critical salt concentration required for the onset of LLPS of 125 µM tick-GRP77. The red-outlined regions in **a** and **b** indicate the condensation regime. **c**, Top left: bright-field image showing a flow-focusing junction of the microfluidic device. The inner stream containing 63 µM tick-GRP77 in Milli-Q water is focused by two streams of 2 M Na_2_HPO_4_. As the three streams flow side by side, GRP condensates are observed to form exclusively at the GRP–salt interface. The condensates wet the channel wall and increase in size over time. **d**, Fluorescence intensity plot, corresponding to the dotted line in **c** (bottom left), shows the intensity profile across the interface of GRP–salt streams. **e**, GRP condensates stuck to the channel walls downstream of the junction are deformed into tear-shaped droplets because of the fluid flow, indicating their liquid-like nature.

Tick saliva is salty and hygroscopic, which helps ticks to absorb moisture to stay hydrated^59^. Additionally, tick saliva contains enzymes such as apyrase and acid phosphatase, which can increase local phosphate concentration through adenosine triphosphate (ATP) degradation^60^. Studies have reported that kosmotropic ions (HPO_4_^2−^, SO_4_^2−^ and so on) facilitate LLPS by having strong bonding interactions with water molecules, thereby decreasing protein solubility and promoting phase separation^61,62^. To test the effect of kosmotropic salts on the tick-GRP77 phase transition, we chose disodium hydrogen phosphate (Na_2_HPO_4_) salt, mimicking the potential inorganic phosphate build-up at the site of the cement cone. Indeed, the addition of 1 M Na_2_HPO_4_ (in 10 mM Tris-Cl, pH 7.4) to 63 µM and 125 µM tick-GRP77 solutions led to instant coacervation (Fig. 4a). We tested several different GRP concentrations and incubation times up to several hours to obtain a phase diagram (Fig. 4a and Supplementary Fig. 9). As can be seen, a short incubation time of 1.5 h at room temperature was enough to form condensates at much lower concentrations (16 µM). Concentrations below 16 µM did not result in condensation even after 5.5 h, and at lower Na_2_HPO_4_ concentrations (0.5 M) we did not observe immediate condensation, but only after several hours of incubation and at high GRP concentrations (>63 µM) (Supplementary Fig. 9). Tick saliva is a crowded environment due to the presence of other biomolecules. We mimicked such a crowded environment with the addition of polyethylene glycol (PEG) molecules. We observed instant phase separation at much lower concentrations of phosphates in crowded conditions (Fig. 4b). For example, 5% wt/vol PEG (8 kDa) led to instant LLPS of 125 µM tick-GRP77 in the presence of 0.5 M phosphate, and further increasing the PEG concentration to 7.5% wt/vol reduced the critical phosphate concentration for phase separation to 0.25 M.

To further clarify the instant nature of the coacervation, we performed flow-focusing experiments using microfluidic devices. A key feature of microfluidic systems is their laminar flow^63^, allowing strictly diffusion-based mixing between the fluid streams. We used a lab-on-a-chip device, allowing the GRP and salt streams to meet and co-flow together without mixing (details are provided in Methods and Supplementary Fig. 10). GRP solution (63 µM in Milli-Q water; 5 mol% OG488-GRP77) was injected into the inner aqueous channel and 2 M Na_2_HPO_4_ solution was injected into the outer aqueous channels, forming two sharp tick-GRP77–Na_2_HPO_4_ interfaces at the junction (Fig. 4c, top left). We observed the immediate formation of tick-GRP77 condensate droplets at the interface, which adhered to the channel walls and continuously increased in size as more and more condensate phase accumulated (Fig. 4c and Supplementary Video 3). A line profile perpendicular to the interface showed two clear fluorescence intensity peaks at the interfaces, clarifying the salt-induced condensation (Fig. 4d). The liquid nature of these condensates became more evident further down the channel where the condensate droplets wetting the channel walls were deformed by the fluid flow into tear-shaped droplets (Fig. 4e).

### Tick-GRP77 condensates undergo a liquid-to-gel transition

**Fig. 5:**
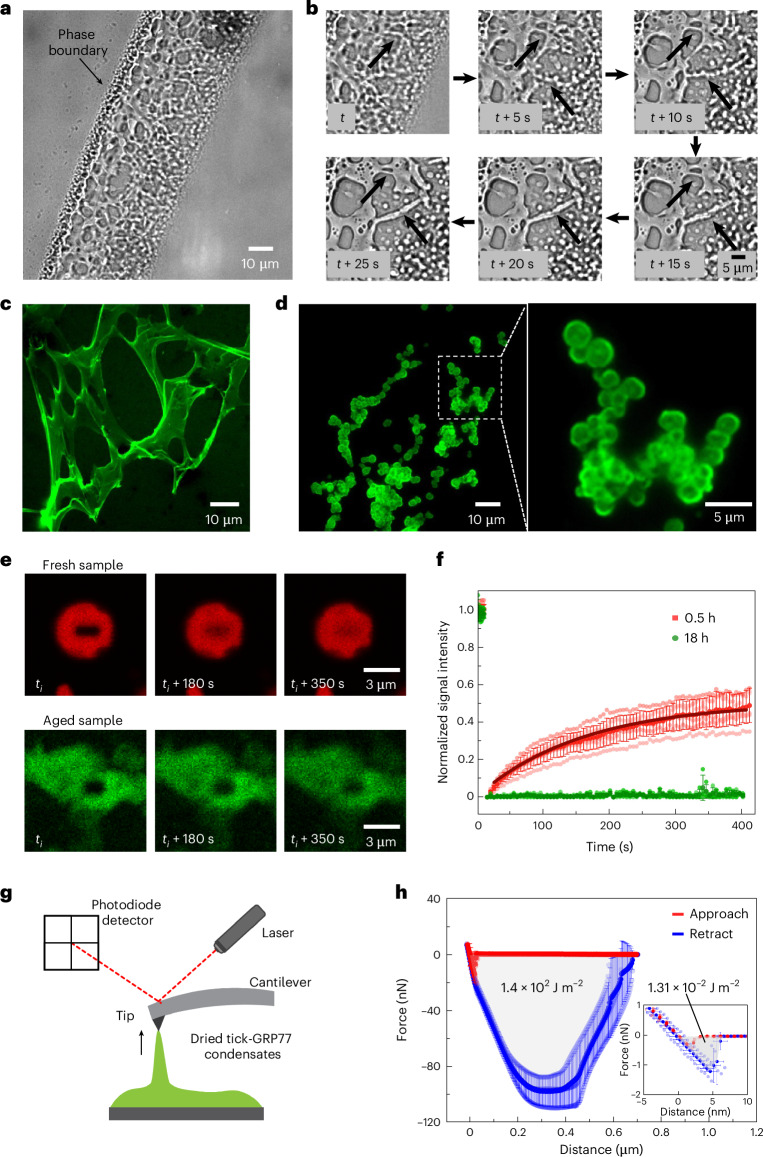
GRP condensates form viscoelastic networks and solid-like aggregates and exhibit adhesive properties. **a**, Droplet evaporation assay of a concentrated tick-GRP77 solution (500 µM in PBS) resulted in the formation of condensate fibres, leading to interconnected gel-like networks. **b**, Time-lapse showing the assembly of the condensates into fibre-like structures. Two such events are indicated by black arrows. **c**, Fluorescence image of an interconnected stretched network formed during an evaporation assay of tick-GRP77 (32 µM in five-times-concentrated PBS) on a PVA-passivated glass slide. **d**, Incubating tick-GRP77 solution (125 µM) in 1 M Na_2_HPO_4_ for 5.5 h at room temperature formed stable condensate clusters. The zoom-in shows physically connected condensates with complete arrest of coalescence. **e**, Time-lapse showing the fluorescence recovery of GRP condensates (125 µM) for fresh (0.5 h) and older (18 h) samples. The condensates were formed in the presence of 1 M Na_2_HPO_4_ salt. **f**, Fluorescence recovery curve showing that the freshly formed condensates (*n* = 6) have a higher fraction of the mobile phase compared with matured GRP condensates (*n* = 4). The solid line shows an exponential fit to the dataset (*R*^2^ = 0.99). Samples in **c**–**f** were doped with 5 mol% OG488-GRP77. **g**, Schematic of adhesion measurements showing the force spectroscopy set-up. **h**, Approach (red) and retract (blue) force–distance curves for the surface coated with tick-GRP77 condensates (125 µM) formed in the presence of 1 M Na_2_HPO_4_ (*n* = 3 different samples). The work of adhesion is four magnitudes higher than that of the negative control (*n* = 3 different samples). The inset shows approach and retract force–distance curves for a non-adhesive tick-GRP77 solution (125 µM) in Milli-Q water acting as a negative control. The plots in **f** and **h** are represented as mean ± s.d., with the dotted curves showing individual datasets. Source data

Natural tick saliva eventually undergoes a liquid-to-solid transition to form a hard cement cone. We observed several instances in our experiments that indicate that LLPS may be an intermediate stage in this transition. Conducting the droplet evaporation assay using a high initial tick-GRP77 concentration (500 µM) on a hydrophobic glass slide not only led to condensate formation, but the formed condensates fused together to form network-like structures (Fig. 5a and Supplementary Video 4). A time-lapse showing an example of fibre formation leading to an interconnected network of tick-GRP77 condensates is shown in Fig. 5b. Along similar lines, a network composed of stretched sheets and fibres adhering to a PVA-coated glass surface was obtained during an evaporation experiment (Fig. 5c). These examples point to the transition of liquid condensates into viscoelastic gel-like networks on solid surfaces.

More solid-like structures were observed during salt-induced coacervation when using a high concentration of tick-GRP77 (125 µM) over longer incubation times (5.5 h). Here, the coalescence of the condensates was clearly arrested, resulting in stable clusters (Fig. 5d). To verify the solidification or ageing of GRP condensates over time, we performed fluorescence recovery after photobleaching (FRAP) experiments (125 µM tick-GRP77 in 1 M Na_2_HPO_4_, pH 7.4; details are provided in Methods). A comparative FRAP study was conducted on freshly prepared condensates (0.5 h after preparation; *n* = 6) and older samples (18 h of incubation; *n* = 4), as shown in Fig. 5e,f (also Supplementary Videos 5 and 6), which revealed two important findings. First, the post-bleaching fluorescence intensity did not show full recovery in both cases, which shows the arrested motion of GRP molecules within condensates already in early stages and their apparent viscoelastic behaviour. Second, fresh samples showed much greater recovery (~49%) compared with the aged samples (~2%). This drastic decrease in the fluorescence recovery of aged samples shows a further reduction in the fraction of mobile phase over time and the transformation from liquid to a solid-like state.

For the fresh sample, we obtained a relaxation time, *τ*_fresh_ = 160 s, with the diffusion coefficient of OG488-GRP77 (*D*_app_) in the order of ~2.2 × 10^−3^ µm^2^ s^−1^ (Methods). We subsequently estimated the condensate viscosity *η* as ~42 Pa s, and the interfacial tension *γ* as ~46 µN m^−1^, the latter similar to the very low values reported for macromolecular liquids^64^ as well as protein condensates^48^. Thus, microscopic and FRAP analysis together demonstrated tick-GRP77 condensates to be highly viscous liquids with ultralow interfacial tension, capable of forming viscoelastic networks and exhibiting ageing over the course of a few hours. This liquid-to-gel transition is highly relevant to tick cement cone formation, which also takes place over several hours^11^.

We further explored whether tick-GRP77 condensates exhibited adhesive properties. For this, we carried out force measurements on air-dried tick-GRP77 condensates (induced in the presence of 1 M Na_2_HPO_4_, pH 7.4) using force spectroscopy (Fig. 5g and Methods). The inset in Fig. 5h shows the force–distance curves obtained on an air-dried aqueous solution of tick-GRP77 without any salts (*n* = 3), that is, in non-coacervating conditions. This acted as a negative control, giving us a base value of work of adhesion of *W*_adh_ = 1.31 × 10^−2^ J m^−2^. On the other hand, *W*_adh_ for the surface coated with tick-GRP77 condensates (*n* = 3) was measured to be 1.4 × 10^2^ J m^−2^, four orders of magnitude higher. These measurements clearly indicate the highly adhesive nature of tick-GRP77 condensates, which could be playing a crucial role in tick adhesion.

### Natural tick saliva shows evidence of protein condensates

**Fig. 6:**
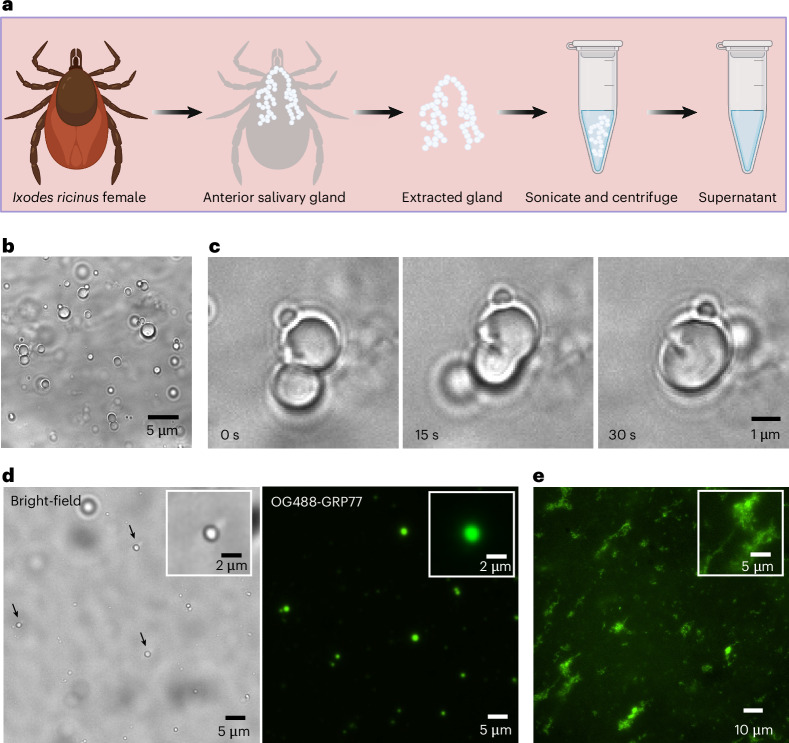
Salivary gland extracts from ticks hint at the presence of protein-rich phase-separated droplets. **a**, Schematic overview showing the extraction of the salivary gland from a non-blood-fed tick (*I. ricinus*). The collected supernatant was used in the following experiments. **b**, Bright-field visualization of the supernatant showing numerous micrometre-sized spherical droplets. **c**, Fusion of two droplets, indicating their liquid nature. **d**, Spiking the supernatant with fluorescently labelled GRP77 (OG488-GRP77) readily led to its partitioning inside the phase-separated droplets (marked with arrows in the bright-field image) indicating their protein-rich nature. **e**, Fluorescence image showing fibre-like structures observed in the supernatant in the presence of 0.75 M Na_2_HPO_4_ with OG488-GRP77 (6 µM), showing strong partitioning in these structures. Insets in **d** and **e** are zoom-ins from the respective images.

All these in vitro experiments encouraged us to further investigate whether natural tick saliva exhibits similar phase-separating behaviour. We collected ticks (*Ixodes ricinus*) from their natural habitat, dissected adult females, and extracted the contents of salivary glands (Fig. 6a, Methods and Supplementary Fig. 11). A denaturing sodium dodecyl sulfate polyacrylamide gel electrophoresis (SDS–PAGE) analysis of the extract showed multiple protein bands in the 10–100 kDa range (Supplementary Fig. 12). We note that the ticks we collected were not blood-fed and thus probably have a lower expression of GRPs compared with blood-fed ticks^21^. Additionally, tick species with long hypostomes, like *I. ricinus*, have a lower level of cement production^65^, and thus probably a lower expression of GRPs^18^. Nonetheless, microscopic visualization of multiple salivary gland extracts (three out of five extractions) revealed numerous micrometre-sized spherical droplets (Fig. 6b). We also recorded a droplet fusion event indicating their liquid nature (Fig. 6c). We ruled out the possibility of these droplets being lipid or fat droplets by confirming that GRP had no affinity to partition inside oil-in-water emulsions (Supplementary Fig. 13). Moreover, subjecting the tick salivary extract to high salt concentrations (0.75–1 M Na_2_HPO_4_, pH 7.4) led to fibre-like structures (Fig. 6e). When we doped the samples with fluorescently labelled OG488-GRP77, we observed strong partitioning (three out of four samples) in these droplets/fibres, hinting at their protein-rich nature (Fig. 6d,e).

These experiments indicate the general likelihood of GRP-rich condensates in the saliva and not particularly tick-GRP77. To emphasize this point, we compared tick-GRP77 with 20 different GRPs identified in tick saliva across nine different species (Supplementary Table 1). Interestingly, the amino-acid composition and patterning of these GRPs show a striking resemblance to that of tick-GRP77. The aromatic amino-acid residues remain prominent (9–20%), and these aromatic residues are also interspersed periodically by non-aromatic units such as glycine and proline. Thus, the tick-GRP77 condensate mechanism that we have shown here could be a more generic path to achieve a homogeneous-to-coacervate-to-solid transition.

## Discussion

We have demonstrated that the glycine-rich protein (GRP; UniProt Q4PME3) present in tick saliva undergoes LLPS via simple coacervation and exhibits ageing behaviour to form gel-like adhesive structures over the course of a few hours. Our results suggest a plausible role of GRPs in cement cone formation, a vital process for ticks to attach to their host. We show that the regularly spaced arginine and aromatic (phenylalanine and tyrosine) residues, separated by glycine-rich regions, are key to the LLPS process via cation–*π* and *π*–*π* interactions.

GRPs are also found in adhesives from various other organisms, with the liquid-to-solid transition crucial for the adhesion process^3,35–37,66^. For instance, the slime proteins of velvet worms undergo LLPS to form sticky fibres^67^, and the mussel foot proteins form condensates that undergo a liquid-to-solid transition^68^. Thus, understanding the phase transitions of tick GRPs may reveal possible common unifying principles in bioadhesive proteins functioning in distinct environments across diverse animal species. Given that secretory proteins are prone to extensive post-translational modifications (for example, α-gal modifications^69^ and phosphorylation^70,71^), their impact is worth investigating. Also worth studying is the possibility of crosslinking, as tick saliva harbours essential enzymes needed for covalent crosslinking, and could assist in the hardening process, as shown in the case of other bioadhesives^11,72^. Finally, given the fact that ticks detach from the host post-feeding^19,73^, a dissolution mechanism of the hardened cement cone will also be worth exploring.

Tick GRPs could potentially be used for the development of medical sealants because of their adhesive and biocompatible properties^11^. Studying tick GRPs may provide insights to manage tick-borne diseases—a major problem worldwide, particularly in developing tropical regions^74^. Developing chemicals that can interfere with protein phase transitions and thus inhibit cement cone formation may provide an effective solution. Anti-tick vaccines are another promising tick control strategy^74–77^, and GRPs have been shown to be immunogenic, making them promising candidates^78–80^. With condensates having great potential as effective drug-delivery systems^81^, testing the potential of GRP-based condensates as anti-tick vaccine agents is another promising future research direction.

## Methods

### Materials

Sodium chloride, potassium chloride, disodium hydrogen phosphate, potassium dihydrogen phosphate, phosphate buffered saline powder, Tris-base, PVA (polyvinyl alcohol, 87–90% hydrolysed, average *M*_w_ 30–70 kDa), bovine serum albumin, β-mercaptoethanol and eucalyptus oil were purchased from Sigma-Aldrich. Food-grade sunflower oil was obtained from Reddy. Precision Plus Protein Dual Xtra Prestained Protein marker, Mini-Protean Tricine Precast Gels 16.5% and 10× Tris/glycine/SDS buffer were purchased from Bio-Rad. Coverslips #1 24 mm × 60 mm were purchased from Corning. SYLGARD184 silicone elastomer and curing agent were purchased from Dow. Silicon wafer was bought from Silicon Materials. Photoresist (EpoCore 10) and photoresist developer (mr-Dev 600) were purchased from Micro Resist Technology GmbH. Microfluidic accessories such as Tygon tubing (1/16-inch outer diameter × 0.02′-inch inner diameter) and the stainless-steel 90° bent polydimethylsiloxane coupler were purchased from Darwin Microfluidics.

### Peptide synthesis and fluorescent labelling

All peptides were synthesized via solid-phase peptide synthesis (SPPS), using two different methods (Boc-based and Fmoc-based SPPS). Details of the synthesis, labelling and characterization are provided in Supplementary section 1 and Supplementary Figs. 14–23. Boc-based SPPS was used for the experiments in Figs. 2, 3c,f, 4a,c–e and 5a–f and Supplementary Figs. 4, 6–9 and 12. Fmoc-based SPPS was used for the experiments in Figs. 3d,e,g, 4b and 5h and Supplementary Figs. 5, 7, 8 and 13.

### In vitro experimentation and microscopic visualization

A stock solution of tick-GRP77 was prepared in Milli-Q water. Unless specified, all the evaporation experiments were performed in PBS (10 mM phosphate buffer, 2.7 mM KCl and 137 mM NaCl at pH 7.4). A 2-µl droplet of protein solution was transferred on a coverslip (24 mm × 40 mm, Corning #1.5) and mounted on a Nikon-Ti2-Eclipse (software, NIS elements) inverted fluorescence microscope equipped with pE-300^ultra^ illumination system. For all the experiments, droplets were visualized using either a Nikon Plan Apo ×100 (numerical aperture (NA) 1.45) oil objective or a Nikon Plan Fluor ×40 (NA 1.30) oil objective. For fluorescent visualization, the sample was doped with 5 mol% OG488-GRP77 and excited using a 482/35-nm excitation filter and a 505-nm dichroic mirror, and the emitted light was collected through a 536/40-nm emission filter (Semrock). The samples were typically excited using 2–5% laser intensity, and time-lapse images were acquired with an exposure of 5–20 ms using a Prime BSI Express sCMOS camera. Confocal microscopy for visualizing the inverted phase and oil-in-water emulsions was recorded using a Nikon C2 laser scanning confocal microscope (software, NIS elements), equipped with a ×60 (NA 1.40) oil immersion objective. The sample was doped with 5 mol% OG488-GRP77 and imaged using a 488-nm excitation laser with excitation filter 525/50, equipped with a 560-nm longpass dichroic mirror and 585/65-nm emission filter. We minimized the illumination intensity (1–2% of 15 mW) to prevent bleaching of the sample.

### Surface functionalization of coverslips

Wherever required, coverslips were coated with 5% wt/vol PVA solution, as described previously^82^. Briefly, the coverslip was plasma-treated for 30 s at 12 MHz (radio frequency mode high) using a plasma cleaner (Harrick plasma PDC-32G). A 10-µl drop of PVA solution was pipetted into the centre of the glass slide and allowed to rest for 5 min, followed by gently removing the PVA by tilting the glass slide. To remove free PVA, the coverslip was gently washed with Milli-Q water. The glass slide was baked at 70 °C for 2 h and stored at room temperature in a clean environment. To make the glass slide hydrophobic, ~20 µl of (tridecafluoro-1,1,2,2-tetrahydrooctyl)trichlorosilane was taken in a glass vial and placed in the desiccator along with the glass slides. The set-up was left under partial vacuum for ~12 h to render the glass slides hydrophobic.

### Droplet fusion experiments

A 2-µl sessile drop of tick GRP solution (32 µM with a 5 mol% fraction of OG488-GRP77; dissolved in PBS pH 7.4) was allowed to evaporate on a PVA-passivated glass slide. Droplet fusion events were recorded using a ×40 oil objective with a time interval of 250 ms (exposure time of 10 ms) on a Nikon Ti2 Eclipse fluorescence microscope. The aspect ratio of the fusing droplets was determined using Fiji (ImageJ) by appropriately thresholding and binarizing the images, and then fitting the droplet boundaries with an ellipse to calculate the aspect ratio ${A}={\frac{\rm{major}\; \rm{axis}}{\rm{minor}\; \rm{axis}}}$, where major and minor axes are the long and short axes of the ellipse, respectively. For analysis of the fusing condensates, the change in aspect ratio with respect to time was plotted in MATLAB and the data were fit to a function of the form ${A}={1}+(A_{0}-1){\rm{e}}^{-\frac{t}{\tau }}$, where *t* is the time, *τ* is the characteristic relaxation time, and *A*_0_ is the initial aspect ratio. Using the relation *τ* ≈ *l*(*η*/*γ*), where *l* is the average diameter of the droplets, *η* is the viscosity and *γ* is the surface tension, the inverse capillary velocity, *η*/*γ*, was calculated^49^.

### Chemical disruption experiments

We used the droplet evaporation assay for both the N terminus and C terminus (50 µM in PBS at pH 7.4) to check the effect of chemicals disrupting specific interactions. To check the involvement of hydrogen bonding, we added 2 M urea solution (0.3 µl to a 1-µl droplet, to a final urea concentration of ~0.5 M) once the condensates were formed. To check the involvement of hydrophobic interactions, we used different concentrations of 1,6-HD (0.5%/1.5%/2.5%/5% wt/vol), adding 0.3 µl 1,6-HD to a 1-µl droplet in each case, once the condensates formed, with final 1,6-HD concentrations of ~15/40/70/140 mM, respectively.

### Microfabrication

The master wafer was prepared according to the previously described ultraviolet lithography method^83^, and the protocol was adjusted to attain a channel height of 20 µm. To prepare the microfluidic device, PDMS and curing agent (SYLGARD184 elastomer) were mixed in a 10:1 weight ratio. The mixture was poured on the master, degassed using a vacuum desiccator, followed by baking at 70 °C for 4 h. The hardened PDMS block was carefully removed, and inlets and outlet holes were punched using a biopsy punch of diameter 0.5 mm (Darwin Microfluidics). The PDMS block was then bonded on a glass coverslip (Corning #1) using a plasma cleaner (Harrick Plasma PDC-32G). The bonded device was baked at 80 °C for 2 h and stored at room temperature. Elveflow pressure controller OB1-MK3 was used to flow GRP and salt solutions 2 M Na_2_HPO_4_ (10 mM Tris-Cl, pH 7.4). Microfluidic reservoir XXS (Darwin Microfluidics LVF-KPT-XXS) was used to load the low-volume (10 µl) GRP samples. The fluid flow was maintained at a constant pressure of 100 mbar and 20 mbar for the inner aqueous (GRP solution) and outer aqueous (Na_2_HPO_4_ solution) channels, respectively.

### FRAP

FRAP experiments were performed on a Leica SP8-SMD microscope (software, LAS-X) and ×63 (NA 1.2) water objective. For bleaching, the region of interest (ROI), of ~1.5-µm length and 0.5-µm breadth, was selected inside condensates of ~5 µm in diameter. The ROI was bleached using 100% laser intensity for 2 s, and recovery of the bleached area was recorded every 5 s for ~6 min. The intensity of the bleached area was normalized using the equation, ${f(t)}={\frac{{I}_{\rm{correct}}(t)-\min ({I}_{\rm{correct}})}{{I}_{\rm{correct}}(0)-\min ({I}_{\rm{correct}})}}$, where ${I}_{\rm{correct}}={C\left(t\right)\times I\left(t\right)}$ and ${C\left(t\right)}={\frac{R(0)}{R(t)}}$. Here, *R*(*t*) and *I*(*t*) indicate the fluorescence intensity of the reference droplet at time *t* and the original fluorescence intensity of the bleached region at time *t*, respectively, while min(*I*_correct_) indicates the minimum value of *I*_correct_, which is obtained immediately after the sample is bleached^84^. The normalized intensity was fitted in Origin using the function ${f\left(t\right)}={A\left(1-{\rm{e}}^{\left(-\frac{t}{\tau }\right)}\right)}$, where *A* and *τ* indicate the amplitude of the recovery and the relaxation time, respectively. The apparent diffusion coefficient (*D*_app_) was calculated using the formula ${{D}_{\rm{app}} \approx \frac{{\omega }^{2}}{{t}_{\left(\frac{1}{2}\right)}}}$, where ${t}_{\left(\frac{1}{2}\right)}$ is the half-life fluorescence recovery and *ω*^2^ is the area of the bleached cross-section. The half-life ${t}_{\left(\frac{1}{2}\right)}$ was calculated using the formula ${t}_{\left(\frac{1}{2}\right)}={\mathrm{ln}\left(2\right){\rm{\tau }}}.$ Droplet viscosity *η* was estimated using the Stokes–Einstein relation ${D}_{\rm{app}}={\frac{{k}_{\rm{B}}T}{6\uppi \eta R}}$, where *k*_B_*T* is the thermal energy scale, and *R* is the hydrodynamic radius of OG488-GRP77 (~2.5 nm for an unfolded 7.8-kDa protein^85^). Finally, plugging the value of *η* into the inverse capillary velocity, *η*/*γ,* led to the estimation of the interfacial tension.

### Adhesion measurements using force spectroscopy

Force–distance measurements were performed on JPK ForceRobot 300, an atomic force microscope specifically designed for force spectroscopy. Silicon wafer was used as a substrate and the aqueous sample containing GRP condensates was dropcast on the wafer and allowed to dry for 1 h. Force spectroscopy was carried out using SCANASYST-AIR silicon-nitride tips with a spring constant of 0.4 N m^−1^. The tip was first approached towards the surface followed by retraction, and the subsequent force–distance curves were measured. Tip retraction led to an adhesive force, allowing the work of adhesion (*W*_adh_) to be measured by dividing the area under the force–distance curve by the tip contact area, considering a 10-nm tip radius. The obtained data were further analysed using JPK SPM Data Processing and plotted in Origin.

### Salivary gland extraction

For tick collection, we selected the Veluwe region in the Netherlands in the months of June and July, when ticks ‘quest’ by climbing up grass and low-lying vegetation and wait for a potential host to pass by, which they will grab and climb on to. We collected ticks belonging to the species *I. ricinus* at different life stages—nymphs, adult males and adult females—from the grass and small shrubs by using a tick-dragging method as previously described^86^. The adult female salivary glands of the Ixodidae groups consist of type I, II and III acini cells^22,59^, among which the type II acini cells are associated with cement formation^22,87^. Female ticks were dissected to isolate their salivary glands according to a previously described protocol^88^, with the exception that the ticks were not blood-fed beforehand. Salivary glands were resuspended in 100 µl of Milli-Q water and their contents were extracted by mechanically disrupting the glands via sonication at 45 kHz for 5 min, followed by debris segregation via centrifugation at 13,000*g* for 10 min. The supernatant was collected in a fresh tube and used for experimentation.

### Statistics and reproducibility

Experiments related to tick-GRP77 phase separation (Fig. 2 and Supplementary Figs. 4–6), mutant studies (Fig. 3 and Supplementary Figs. 7 and 8), material properties (Fig. 5) and tick saliva (Fig. 6 and Supplementary Figs. 1, 12 and 13) were repeated at least three times with similar results. Experiments relating to phase diagrams and microfluidics (Fig. 4 and Supplementary Figs. 9 and 10) were repeated twice. The droplet fusion shown in Fig. 6c is a singular observation.

### Reporting summary

Further information on research design is available in the Nature Portfolio Reporting Summary linked to this article.

## Online content

Any methods, additional references, Nature Portfolio reporting summaries, source data, extended data, supplementary information, acknowledgements, peer review information; details of author contributions and competing interests; and statements of data and code availability are available at 10.1038/s41557-024-01686-8.

## Supplementary information

Supplementary Information Peptide synthesis and labelling, Supplementary Figs. 1–23 and References.

Reporting Summary

Supplementary Video 1 Evaporation of a 2-µl sessile droplet of 32 µM tick-GRP77 (containing 5 mol% OG488-GRP77) in PBS on a glass slide. The coffee ring effect gradually increases the protein concentration at the droplet boundary inducing the formation of tick-GRP77 condensates.

Supplementary Video 2 Coalescence of two tick-GRP77 condensates. Condensates were obtained by evaporating a 2-µl sessile droplet of 32 µM tick-GRP77 (containing 5 mol% OG488-GRP77) in PBS on a PVA-coated glass slide.

Supplementary Video 3 Flow-focusing a stream of 63 µM of tick-GRP77 (containing 5 mol% OG488-GRP77) with two co-flowing 2 M Na_2_HPO_4_ streams leads to the formation of tick-GRP77 condensates at the interfaces of protein–salt streams. The condensates get deformed due to the flow-induced shear.

Supplementary Video 4 Evaporation of a 2-µl sessile droplet with high concentration of tick-GRP77 (500 µM) in PBS on a hydrophobic glass slide led to the formation of an interconnected viscoelastic network of condensates.

Supplementary Video 5 Time-lapse showing the fluorescent recovery after photobleaching of a fresh (0.5-h-old) tick-GRP77 condensate. A small region inside the condensate (obtained by exposing 125 µM tick-GRP77, containing 5 mol% OG488-GRP77, to 1 M Na_2_HPO_4_ solution) was bleached and showed ~50% recovery of fluorescence intensity within a time span of 5 min.

Supplementary Video 6 Time-lapse showing the fluorescent recovery after photobleaching of an aged (18-h old) tick-GRP77 condensate. A small region inside the condensate (obtained by exposing 125 µM tick-GRP77, containing 5 mol% OG488-GRP77, to 1 M Na_2_HPO_4_ solution) was bleached and showed virtually no recovery of fluorescence intensity within a time span of 5 min.

Supplementary Table 1 Comparison of tick-GRP77 with 20 different GRPs identified in the tick saliva across nine different species. The molecular weights of these GRPs range between 7–88 kDa. In addition to the high glycine content, the amino-acid composition and patterning of these GRPs show striking resemblance to that of GRP77. The aromatic amino-acid residues remain prominent (minimum 9% and as high as 20%) and these aromatic residues are also interspersed periodically (typically every 3 to 10 amino acids) by non-aromatic units such as glycine and proline.

Supplementary Data Source data showing the onset of coacervation for different tick-GRP77 concentrations (Supplementary Fig. 5).

## Source data

Source Data Fig. 2 This file contains source data for droplet fusion measurements.

Source Data Fig. 5 This file contains source data for FRAP and atomic force spectroscopy-based adhesion measurements.

## Data Availability

All data supporting the findings of this study are available within the paper, its Supplementary Information, source data and on figshare^89^. Source data are provided with this paper.

## Code availability

The data were analysed using standard software plugins whenever appropriate, as described in Methods. No custom code was written.
