# Supplementary material for: Phase separation and ageing of glycine-rich protein from tick adhesive
Source: Nat Chem. 2024 Nov 29;17(2):186–97. doi: 10.1038/s41557-024-01686-8 (PMC11794139; doi:10.1038/s41557-024-01686-8)
Supplement: Supplementary file 1 — Peptide synthesis and labelling, Supplementary Figs. 1–23 and References. [file 41557_2024_1686_MOESM1_ESM.pdf]

nature chemistry

Supplementary information https://doi.org/10.1038/s41557-024-01686-8

Phase separation and ageing of glycine-rich protein from tick adhesive

In the format provided by the authors and unedited

Table of contents

Section 1: Peptide synthesis and labelling (Page no. 2-5).

Section 2: Supplementary figures (Page no. 6-28)

Section 3: References (Page no. 29)

Section 1: Peptide synthesis and labelling

All peptides were synthesized via solid-phase peptide synthesis (SPPS), using two different methods (Boc- and Fmoc-based SPPS).

Boc-based SPPS

All amino acids and 4-(hydroxymethyl)phenylacetamidomethyl (PAM) resins were obtained from Bachem (Bubbendorf, Switzerland). Serine (Ser), glycine (Gly) and cysteine (Cys) protected with an acetamidomethyl (Acm) group were obtained from the Peptide Institute INC (Osaka, Japan). Cys protected with a MeBzl group was from Iris Biotech (Marktredwitz, Germany). N,N-dimethylformamide (DMF), N,N-diisopropylethylamine (DIPEA), dichloromethane (DCM), and acetonitrile (MeCN) were supplied by Biosolve (Valkenswaard, The Netherlands) and 2-(6-Chloro-1-H-benzotriazole-1-yl)-1,1,3,3-tetramethyluronium hexafluorophosphate (HCTU) was from Peptide International (Louisville, United States). Trifluoroacetic acid (TFA) and ascorbic acid were obtained from VWR chemicals (Leuven, Belgium). Hydrogen fluoride (HF) was purchased from Gerling Holz & Co (Hamburg, Germany). Guanidine (Gnd-HCl) and sodium hydroxide (NaOH) were obtained from Merck-Millipore (Burlington, United States). The radical initiator VA-044 was obtained from Wako Chemicals (Richmond, United States).

The N- (H_2N-APAEEAKPAE AGDEKKDVEG RIGYGGPGFG GG-MPAL) and C- (H_2N-AFGSGFNRGG SFGVGAHGNQ YGQGGFEIQP GRQQPSCVRQ HPNLR-OH) terminal peptide fragments as well as the ΔFY mutant (H_2N-AAGSGANRGG SAGVGAHGNQ AGQGGAEIQP GRQQPSCVRQ HPNLR-OH) were synthesized on 0.10 mmol scale. 0.18 g of resin was used for both syntheses, with PAM polystyrene resin with preloaded leucine (Leu) or arginine (Arg) for the N- and C-terminal peptide fragments, respectively. The first alanine residue (Ala) of the C-terminal peptide fragment was replaced by a cysteine residue (Cys) (A33C mutation) to enable native chemical ligation (NCL). Each amino acid was activated with 0.5 M HCTU in DMF and DIPEA before coupling. The coupling time for all amino acids was 10 minutes, except for serine (Ser), threonine (Thr), Arg, and asparagine (Asn) for which the coupling time was 20 minutes. For glycine (Gly), the coupling time was also set to 20 minutes for the N-terminal peptide fragment, whereas 10 minutes double couplings done for the C-terminal peptide fragment. After each coupling, the resin was washed with DMF, then treated with TFA two times for 1 minute and washed again with DMF. After glutamine (Gln) coupling (C-terminal peptide fragment synthesis), the resin was washed with DCM as well as before and after TFA treatment to prevent intramolecular pyrrolidone formation. For thioester synthesis at the N-terminal peptide fragment, 3-mercaptopropionic acid (MPA) was coupled via a Leu residue and subsequent trityl group-deprotection was performed with a 95%/2.5%/2.5% TFA/Triisopropylsilane (TIS)/H_2O mixture. The N- and C-terminal peptidyl fragments

(495 mg and 440 mg, respectively) were deprotected and cleaved from the solid-phase by anhydrous HF treatment for 1h at 0 °C using 4% v/v p-cresol as scavenger. The peptides were precipitated in ice-cold diethyl ether, dissolved in a MeCN/H_2O mixture containing 0.1% TFA, and lyophilized. To obtain the full tick-GRP77, the following three steps were performed.

Native chemical ligation

NCL of the unprotected synthetic peptide segments was performed as follows: 0.1 M TRIS buffer, pH 8, containing 6 M Gnd-HCl was added to dry peptides yielding approximately 10 mg/mL of peptide fragments. Subsequently, 1% v/v benzylmercaptan and 1% v/v thiophenol were added. The ligation reaction was performed in a heating block at 37 °C and the mixture was vortexed periodically to equilibrate the thiol additives. Reaction progress was analyzed with UPLC-MS. After the reaction was complete, thiophenol was removed by diethylether extraction of the reaction mixture (3x).

Desulfurization

Desulfurization was directly performed after the native chemical ligation reaction in order to convert the first Cys residue of the C-terminal peptide fragment into an Ala residue and thus to obtain the original GRP sequence. Desulfurization buffer was prepared by dissolving tris(2-carboxyethyl)phosphine (TCEP; 250 mM) in 5 mL 0.1 M TRIS buffer, pH 8, containing 6 M Gnd-HCl. The pH of the desulfurization buffer was adjusted to 7 by adding solid NaOH. Then, reduced L-glutathione (GSH; 40 mM) was added to 1 mL of this desulfurization buffer. 250 µL of this solution were added to the reaction mixture and VA-044 (6.25 mM) was added. The reaction was performed at 37 °C and the reaction progress was monitored using UPLC-MS until the product was observed and the starting material could no longer be observed. Analytical HPLC was performed to purify GRP by using a C18 column (150 mm x 4.6 mm) connected to a Prostar HPLC (Varian).

Acm deprotection

To remove the acetamidomethyl (Acm) protecting group of the cysteine residue in the C-terminal peptide fragment, the peptide was dissolved at a 2 mM concentration in 0.1 M TRIS buffer, pH 7.25, containing 6 M Gnd-HCl. Then, 10 eq. Pd-Cl_2 were added and the deprotection progress at 37 °C was monitored using UPLC-MS. After reaction completion, the formed Pd-complex was reduced with 20 mM dithiothreitol (DTT) for 1 hour at 37 °C. Subsequently, the reaction mixture was purified on a C4 column (Vydac, 150 mm x 4.6 mm) with an appropriate gradient on the analytical HPLC system as described above.

Fluorescent labelling

0.5 mg (0.064 µmol) tick-GRP77 was dissolved in 250 µl 0.1 M TRIS buffer containing 6 M Gnd.HCl, pH 7.25. 200 µg (6.5 eq) maleimide-OG488 was added. The reaction was performed at RT and reaction progression was analyzed on UPLC-MS. After 1 h, the reaction mixture was purified on analytical HPLC (C18) using the following time (min)-buffer (%B) parameters: 0-10; 5-10; 10-20; 20-20; 50-50. Buffer A is 0.1% TFA in H_2O; buffer B is 0.1% TFA in MeCN/H_2O (9/1, v/v).

<u>Fmoc-based SPPS</u>

All amino acids and carboxyamidomethyl (Cam) ester (OCam) were obtained from GL Biochem. OxymaPure, 2-(1H-benzotriazol-1-yl)-1,1,3,3-tetramethyluronium hexafluorophosphate (HBTU), and 4-dimethylaminopyridine (DMAP) were purchased from Iris Biotech, while TIS was obtained from TCI chemicals. Tricine and TCEP were obtained from Sigma Aldrich. DMF, DCM, diethylether, MeCN, N,N'-diisopropylcarbodiimide (DIC), DIPEA, and TFA were supplied by BioSolve.

Peptides (tick-GRP77: amine fragment – GFNR GGSFGVGAHG NQYGQGGFEIQPGRQQPSCV RQHPNLR and ester fragment – APAEEAKPAE AGDEKKDVEG RIGYGGPGFG GGAFGS-OCam-L; C-terminus: H_2N-AFGSGFNRGG SFGVGAHGNQ YGQGGFEIQP GRQQPSCVRQ HPNLR; ΔR mutant: H_2N-AFGSGFNAGG SFGVGAHGNQ YGQGGFEIQP GAQQPSCVAQ HPNLA) were synthesized via Fmoc-SPPS on a microwave assisted peptide synthesizer (CEM liberty lite) using a Rink-amide resin with DIC/Oxyma chemisty. The Cam ester was introduced by incubating the resin with 2 eq. Fmoc glycolic acid, 2 eq. HBTU, 2 eq. OxymaPure, and 4 eq. DIPEA for 45 min at room temperature[1]. After fluorenylmethoxycarbonyl (Fmoc) deprotection of the glycolic acid, the ester bond was formed by incubation of 4 eq. Ser, 0.4 mM DMAP and 6 eq. DIC to the resin for 45 min at room temperature. After completion, the peptides were cleaved from the resin (95% TFA; 2.5% TIS; 2.5% H_2O) and purified using RP-HPLC on a C18 column (XBridge C18 5 µm OBD 30x100 mm). Finally, the product was lyophilized (Christ alpha 2-4 LSCbasic) and checked for purity via HPLC/MS (Agilent 1100, LC-MSD SL). To obtain the full tick-GRP77 from amine and Cam ester fragments, the following step was performed.

Ligation

Peptides were dissolved in Tricine buffer (200 mM, pH 8.3, 4 mM TCEP) to a concentration of 1 mM ester fragment, 2 mM amine fragment and 20 µM omniligase-1[2]. The reaction was performed at room temperature for 4 hours and followed via RP-HPLC/MS (Agilent 1100, LC-MSD SL) using an MeCN/H_2O gradient on a C-18 column (Phenomenex 5µm EVO C18 100 Å 150 x 4.6 mm). After completion of the ligation, the product was purified by preparative HPLC (Waters 2545, 2998, Aquity QDA) using a

H_2O/MeCN gradient on a C18 column (XBridge C18 5 µm OBD 30x100 mm). Finally, the product was lyophilized (Christ alpha 2-4 LSCbasic) and checked for purity via HPLC/MS (Agilent 1100, LC-MSD SL).

Peptide mass was verified using mass spectrometry (Supplementary Figures 14-20) and the purity was checked using HPLC (Supplementary Figures 21-23).

Section 2: Supplementary Figures

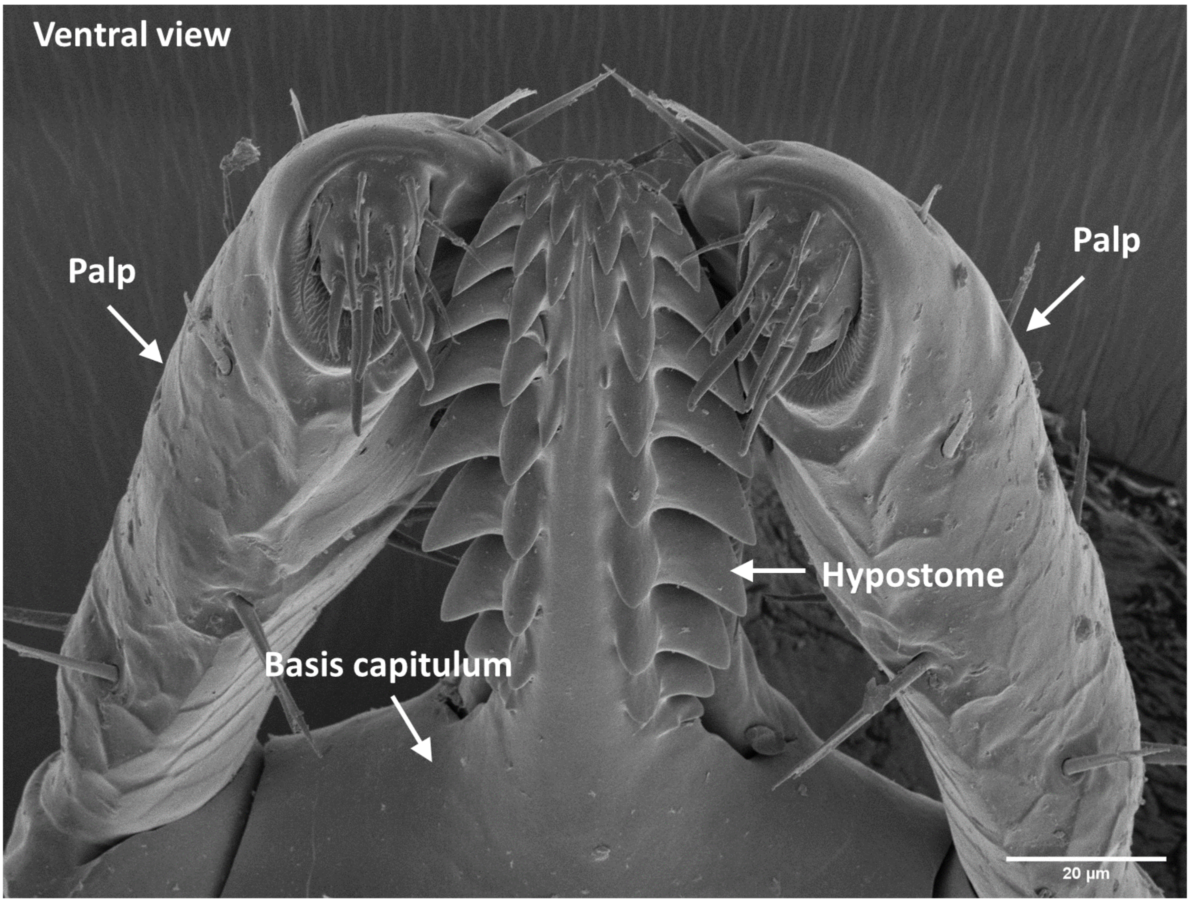

Supplementary Figure 1. Tick mouth parts. Electron microscopy of tick mouth parts visualized from the ventral side.

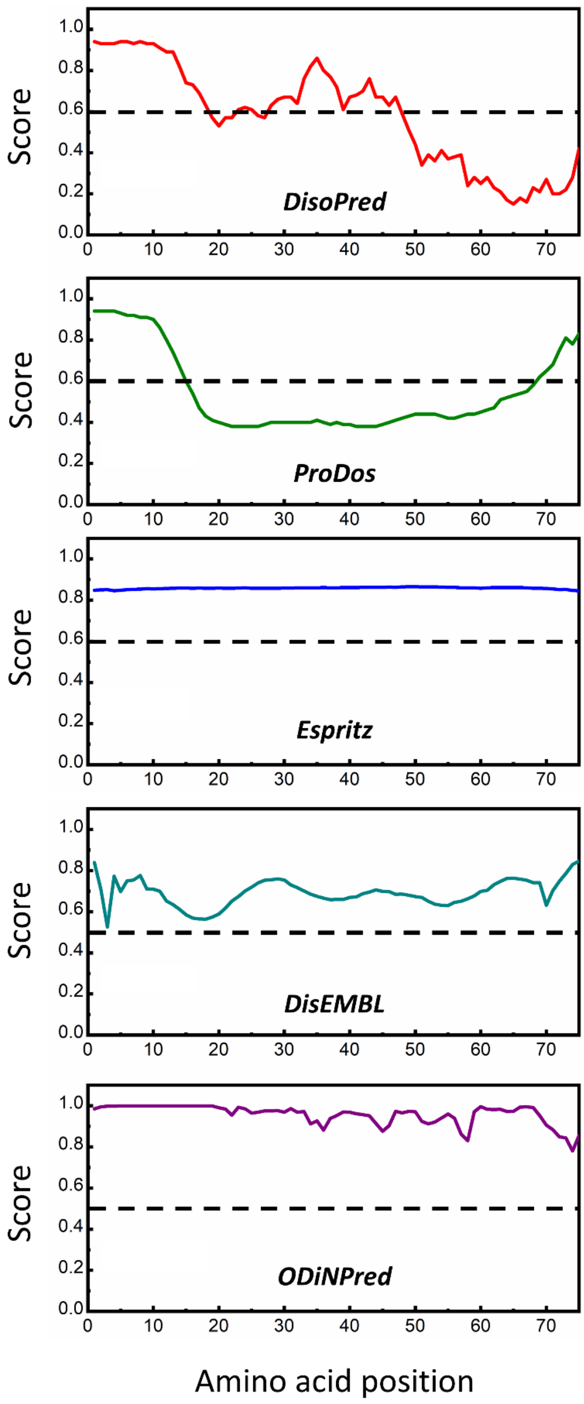

Supplementary Figure 2. Disorder prediction in tick-GRP77. Multiple softwares predict that the tick-GRP77 is highly disordered, or at least have prominent disordered regions. DisoPred[3] predicts that the N-terminus has a high disorder and ProDos[4] suggests both N- and C-terminus as disordered regions. Espritz[5], DisEMBL[6] and ODiNPred[7] predict the entire tick-GRP77 to be completely disordered.

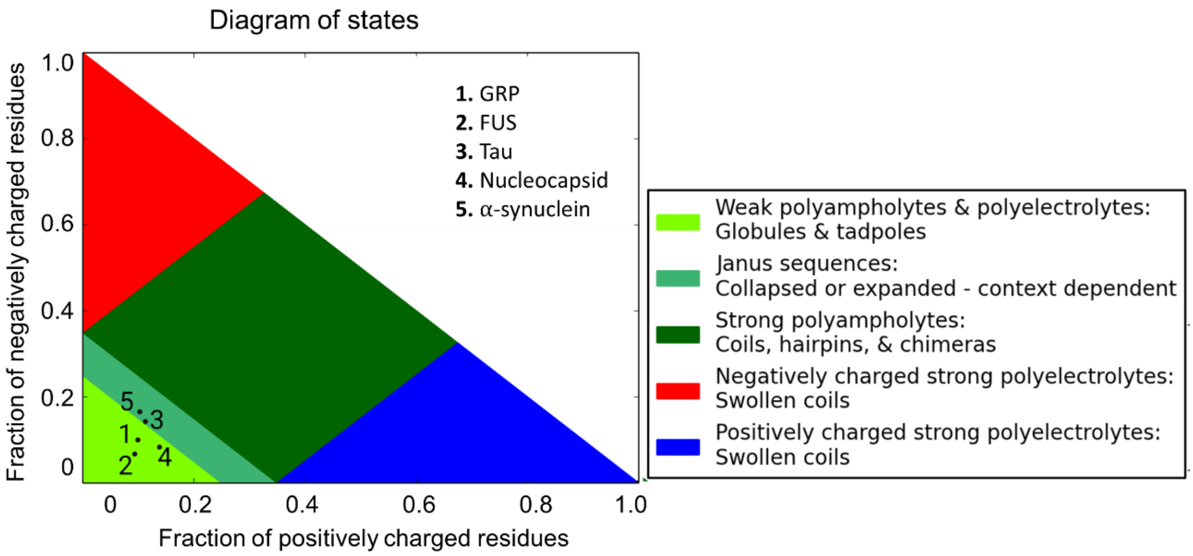

Supplementary Figure 3. Tick-GRP77 falls in the same region within the diagram of states as many well-characterized condensate-forming proteins. Diagram of states indicating tick-GRP77 as weak polyampholytes and polyelectrolytes and falls in close vicinity of FUS, tau, nucleocapsid protein, and α-synuclein, all of which are known to phase separate.

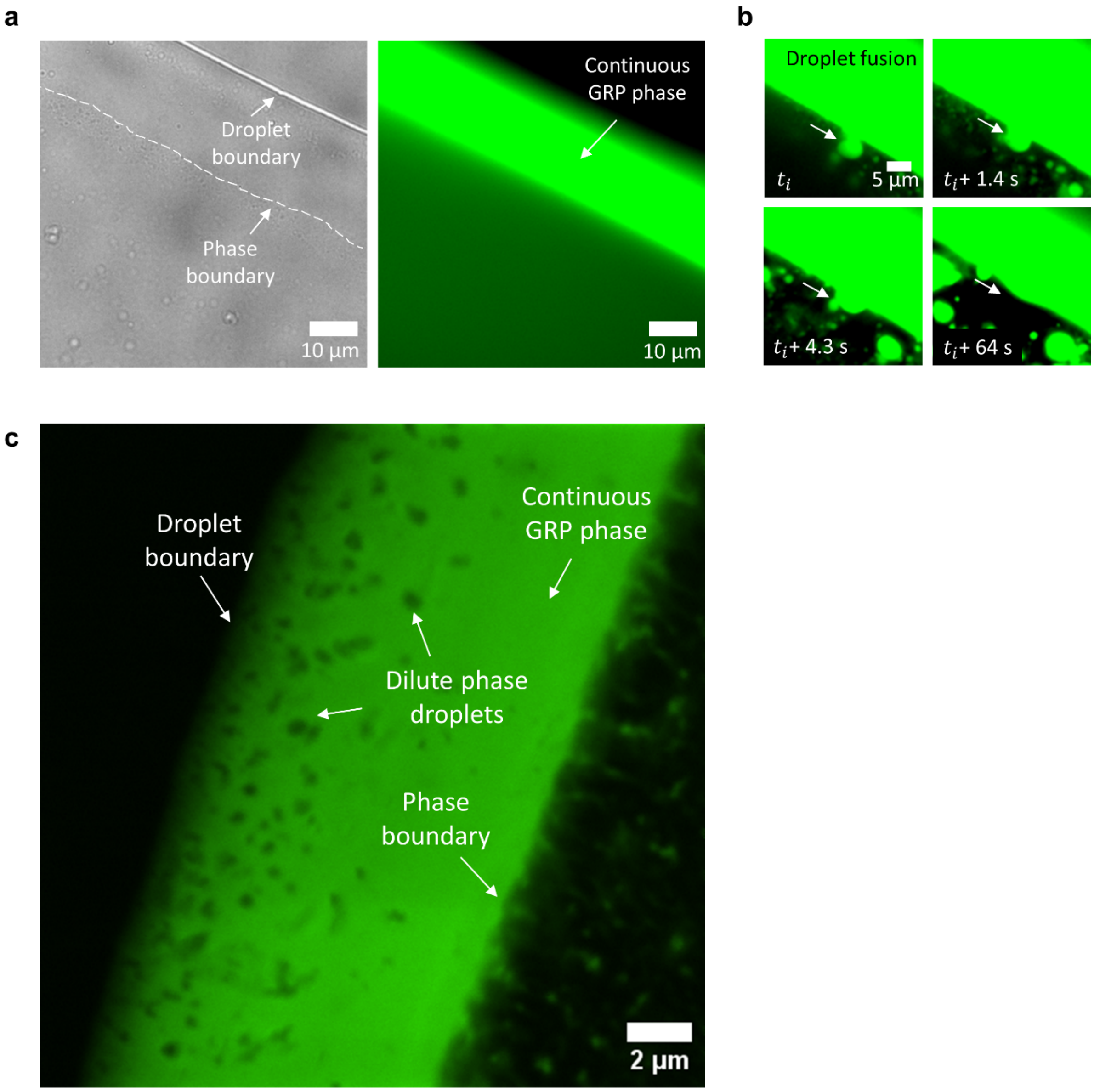

Supplementary Figure 4. The region between the droplet boundary and the rim is a tick-GRP77-rich condensate phase. (**a**) Bright-field (left) and corresponding fluorescence image (right) showing the tick-GRP77-rich nature of this region (32 µM starting concentration). (**b**) An example of tick-GRP77 condensate fusing with the continuous tick-GRP77-rich phase. (**c**) Confocal microscopy showing tick-GRP77-depleted aqueous droplets within the inverted phase formed by evaporation assay (125 µM starting concentration). In all cases, tick-GRP77 was present in PBS and 5 mol% OG488-GRP77 was added for fluorescence visualization.

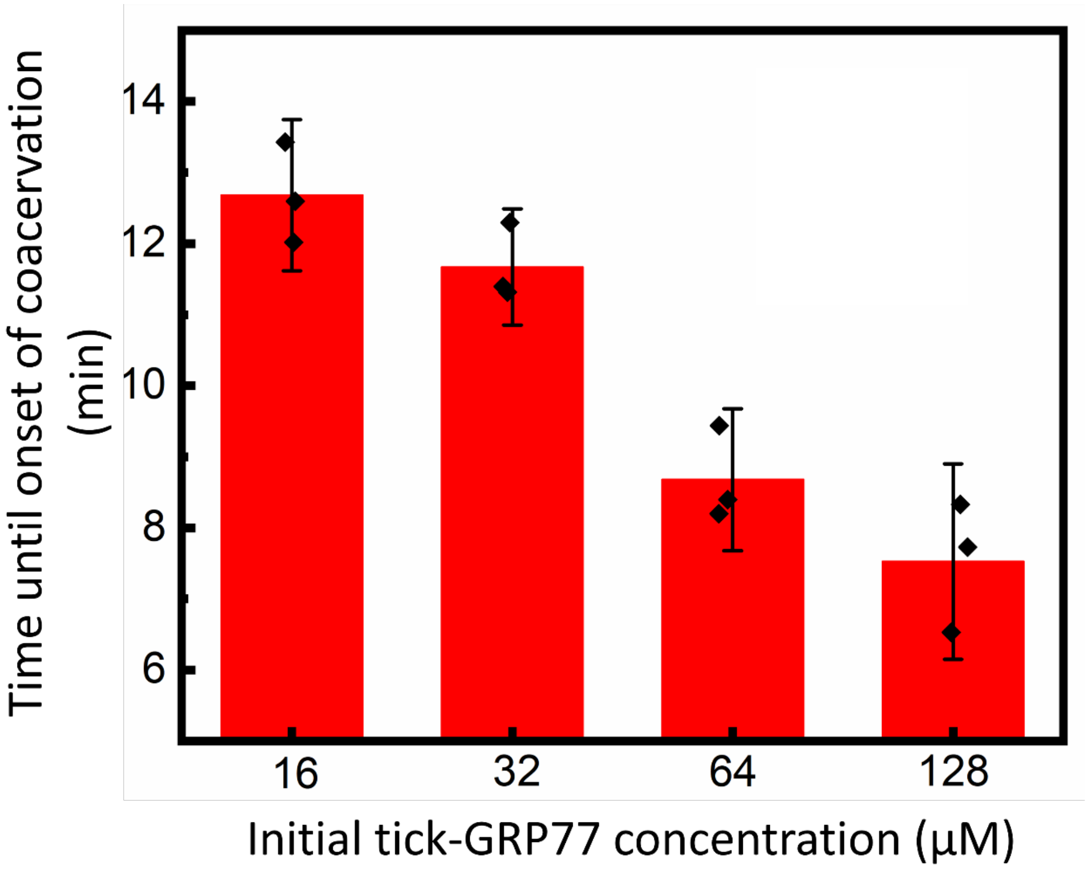

Supplementary Figure 5. Onset of coacervation is influenced by the starting tick-GRP77 concentration. The time required for the onset of coacervation is steadily reduced as the initial tick-GRP77 concentration is increased (n = 3 different samples in each case). The values were recorded during droplet evaporation assay in PBS. Data are represented as mean ± s.d., with the dots showing individual values.

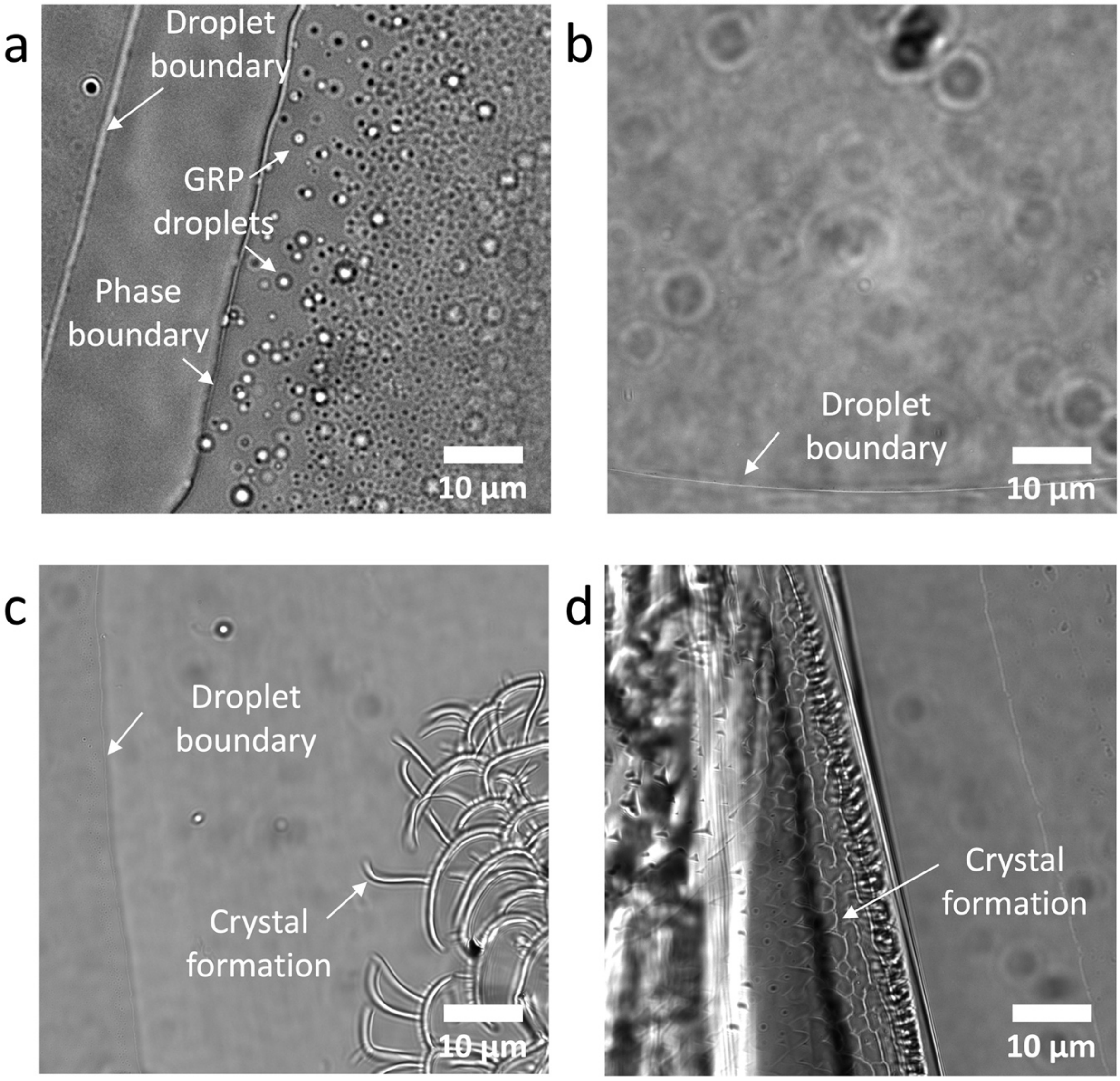

Supplementary Figure 6. The formed condensates are specific to tick-GRP77 and need salts for their formation. (**a**) Evaporation of tick-GRP77 (32 µM) in the presence of 140 mM NaCl resulted in the formation of condensates. (**b**) On the contrary, evaporation of tick-GRP77 (32 µM) dissolved in pure water did not lead to phase separation. (**c**) Evaporation of globular protein solution, bovine serum albumin (127 µM in PBS) eventually led to the formation of salt crystals without any phase separation. (**d**) Similarly, just a PBS solution did not form condensates but salt crystals.

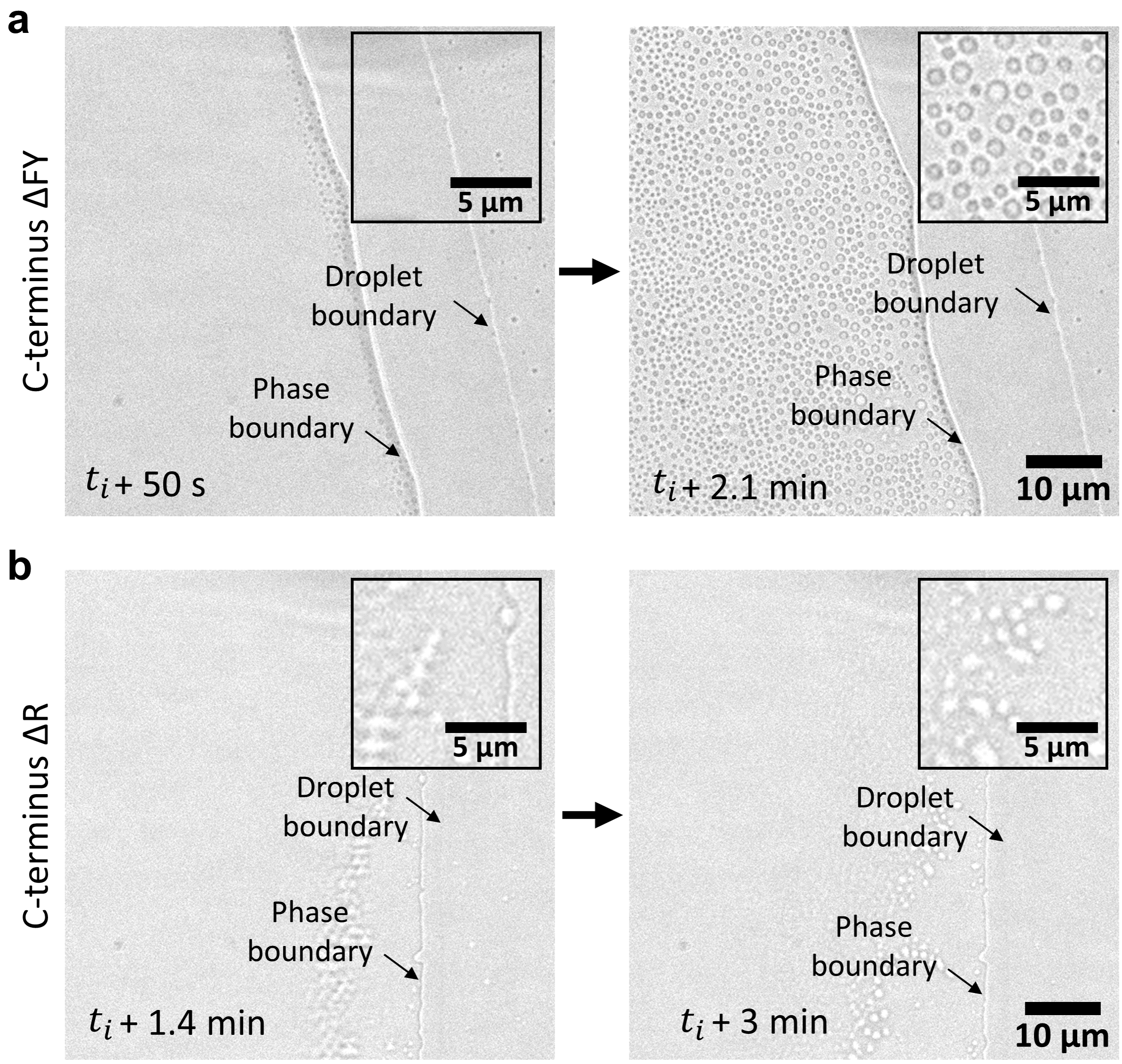

Supplementary Figure 7. **Evaporation assays of 100 µM C-terminus mutants**. (**a**) Higher initial concentration of ΔFY mutant showed slightly faster initiation of coacervation ($t_i \approx$ 6.5 min) and proceeded with appreciable coacervation. (**b**) Higher initial concentration of ΔR mutant accelerated the initiation of coacervation ($t_i \approx$ 4.8 min) but only negligible amount of coacervates appeared near the rim over time. The starting concentration of all the samples was 100 µM in PBS.

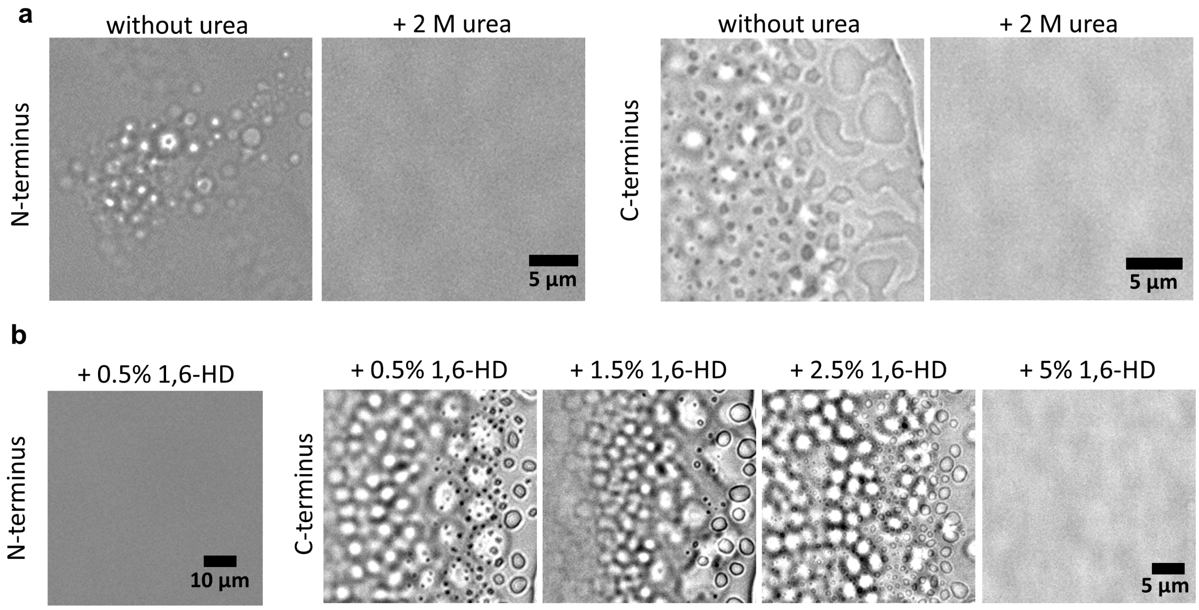

Supplementary Figure 8. Hydrogen bonding and hydrophobic interactions are involved in phase separation of tick-GRP77. (**a**) Both N- and C-terminus condensates formed via droplet evaporation assay dissolved upon addition of urea (≈0.5 M final concentration) indicating an active role of hydrogen bonding in LLPS formation. (**b**) N-terminus condensates formed via evaporation dissolved upon addition of 0.5% w/v 1,6-HD (≈15 mM final concentration). On the contrary, C-terminus condensates remained unaffected in presence of 1.5% w/v 1,6-HD (≈40 mM final concentration), and completely dissolved only at 5% w/v 1,6-HD (≈140 mM final concentration), indicating a prominent role of hydrophobic interactions compared to N-terminus condensates. Starting concentrations for both termini were 50 µM in PBS for all the experiments.

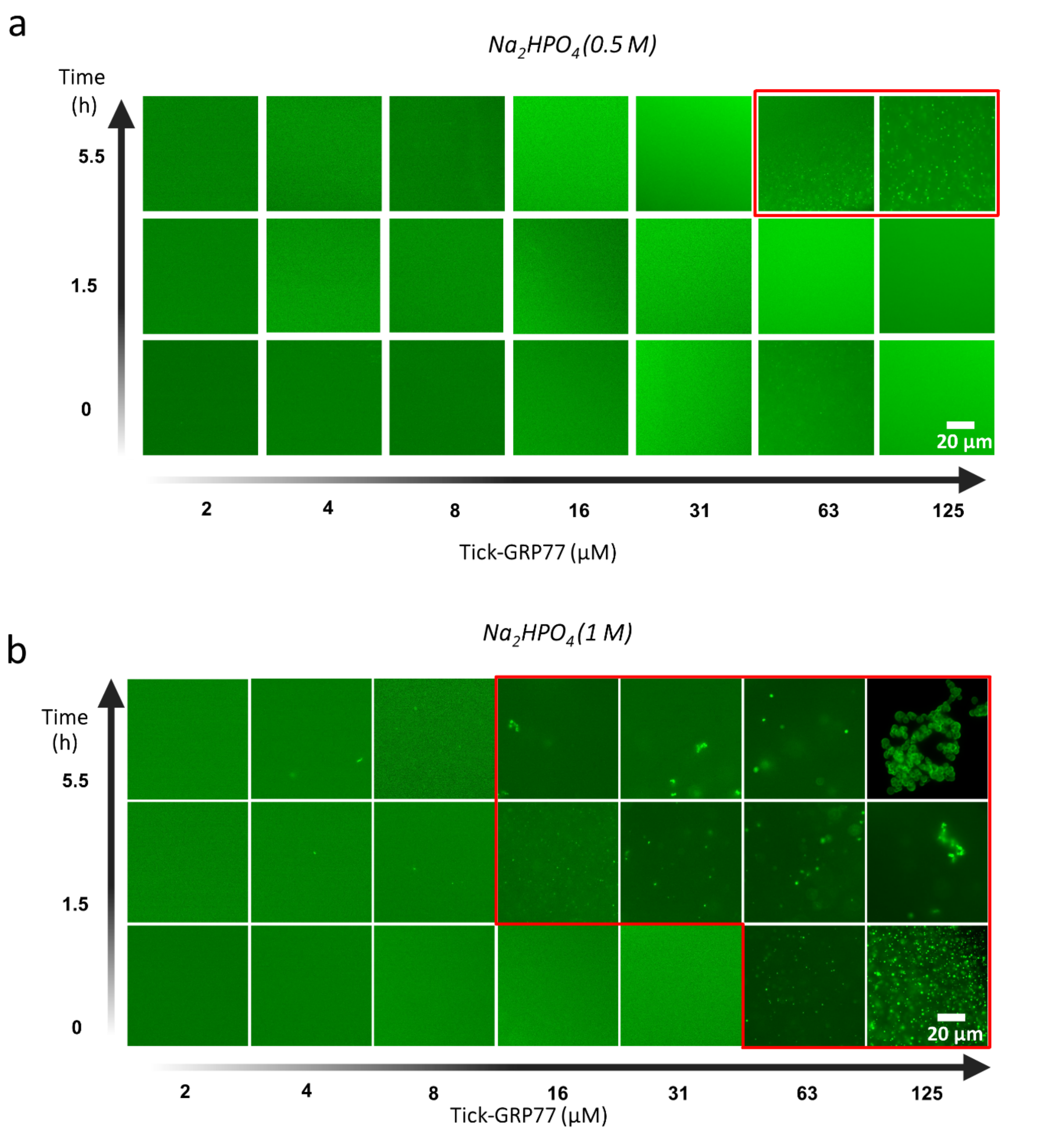

Supplementary Figure 9. Tick-GRP77 coacervation phase diagram as a function of protein concentration and incubation time in Na_2HPO_4 solution. (**a**) Incubation of tick-GRP77 (2–125 µM) at 0.5 M Na_2HPO_4 did not lead to instant condensate formation. At 63 µM and above, condensates formed after 5.5 hour of incubation. (**b**) On the contrary, presence of 1 M Na_2HPO_4 led to instant coacervation of tick-GRP77 at 63 µM and above. Incubation for 1.5 hours also led coacervation down till 16 µM tick-GRP77.

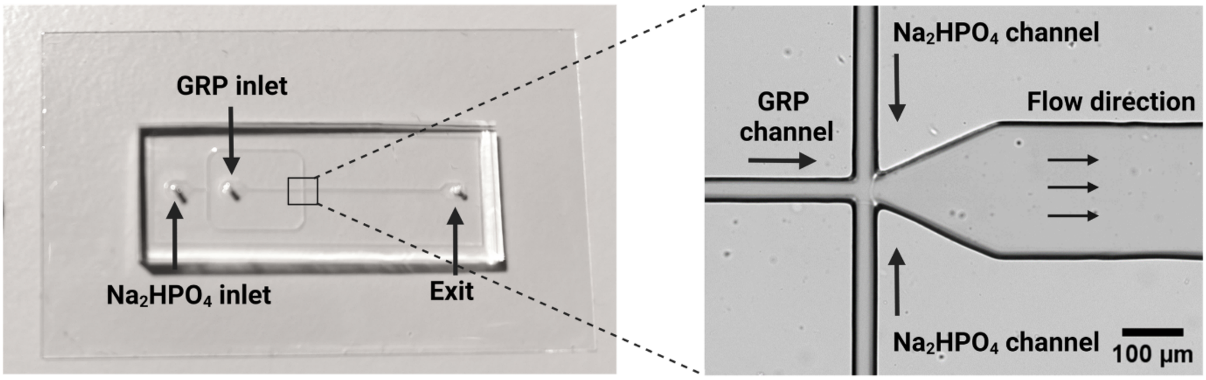

Supplementary Figure 10. Microfluidic device used in flow-focusing experiments. Bright-field images showing PDMS-based lab-on-a-chip device with a zoom-in showing the flow-focusing junction.

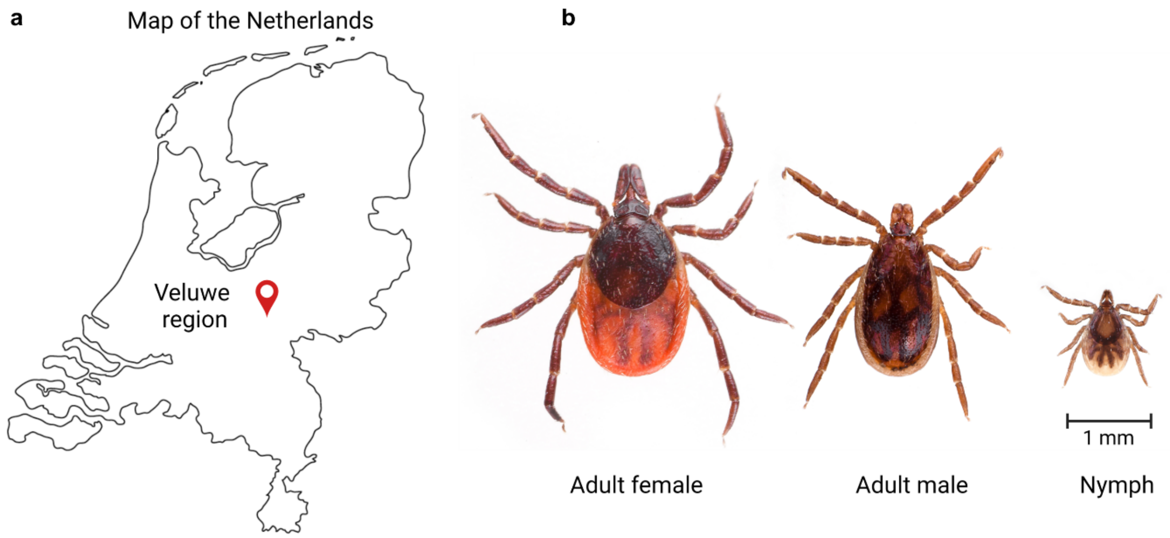

Supplementary Figure 11. Tick Collection. (**a**) Location of tick collections in the Netherlands. (**b**) Ticks of species *Ixodes ricinus* at different life stages were collected.

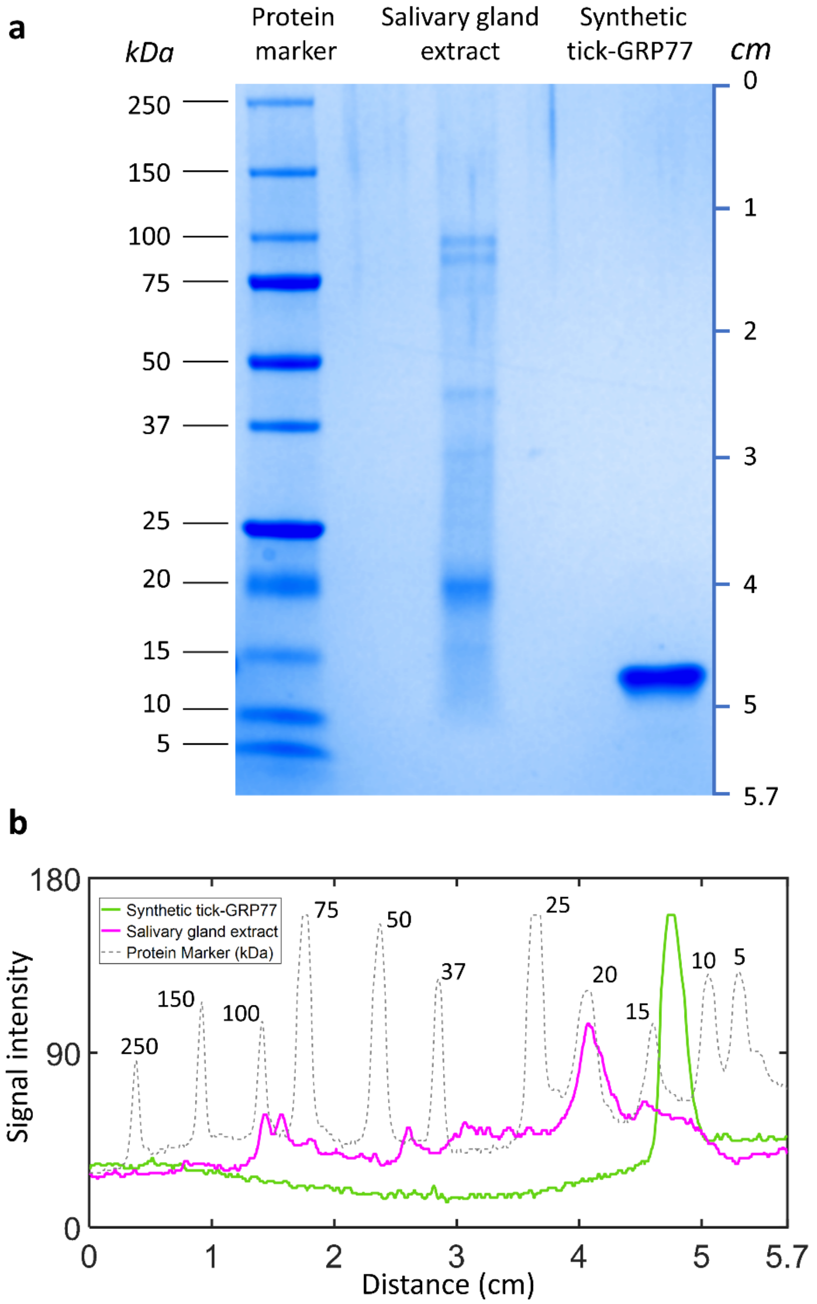

Supplementary Figure 12. Protein gel electrophoresis of tick salivary gland extract and tick-GRP77. (**a**) Denaturing SDS-PAGE of the tick salivary gland extract (middle lane) isolated from a single adult female *Ixodes ricinus* showed proteins of varying molecular weights ranging from 10 to 100 kDa. Some prominent bands were observed, such as one corresponding to 20 kDa protein marker (first lane). Synthetic tick-GRP77 (last lane) showed intense single band between 10 and 15 kDa. (**b**) Protein signal intensity plot versus protein migration distance shows single band of high intensity for synthetic tick-GRP77 whereas tick salivary gland extract shows maximum signal intensity at 20 kDa in addition to low signal dispersed between 10 and 100 kDa. Protein marker intensities were used as reference.

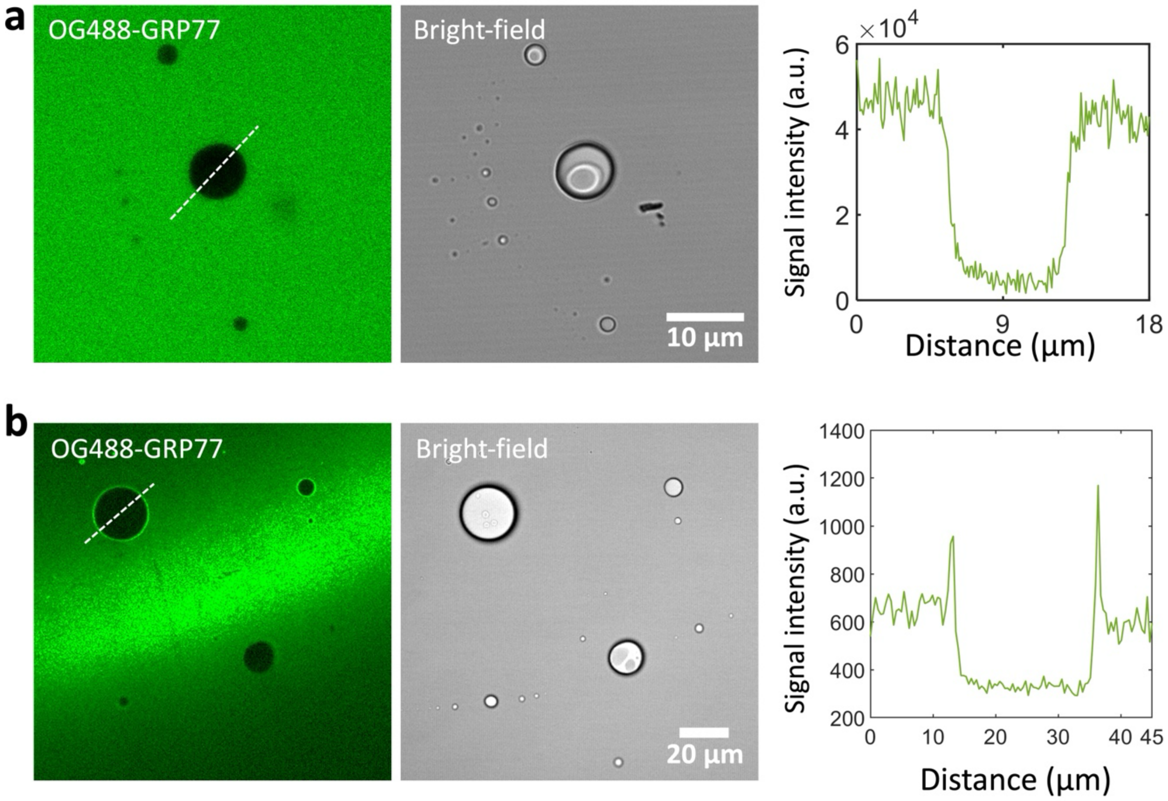

Supplementary Figure 13. Tick-GRP77 does not partition in hydrophobic oil-in-water emulsions. (**a**) OG488-GRP77 did not partition in sunflower oil droplets. (**b**) OG488-GRP77 also did not partition in eucalyptus oil droplets but showed some preference to remain at the oil-water interface. In both the panels, the line graphs correspond to the dotted lines and show the exclusion of OG488-GRP77 from the oil droplets. In all cases, the final concentration of OG488-GRP77 was 2 µM.

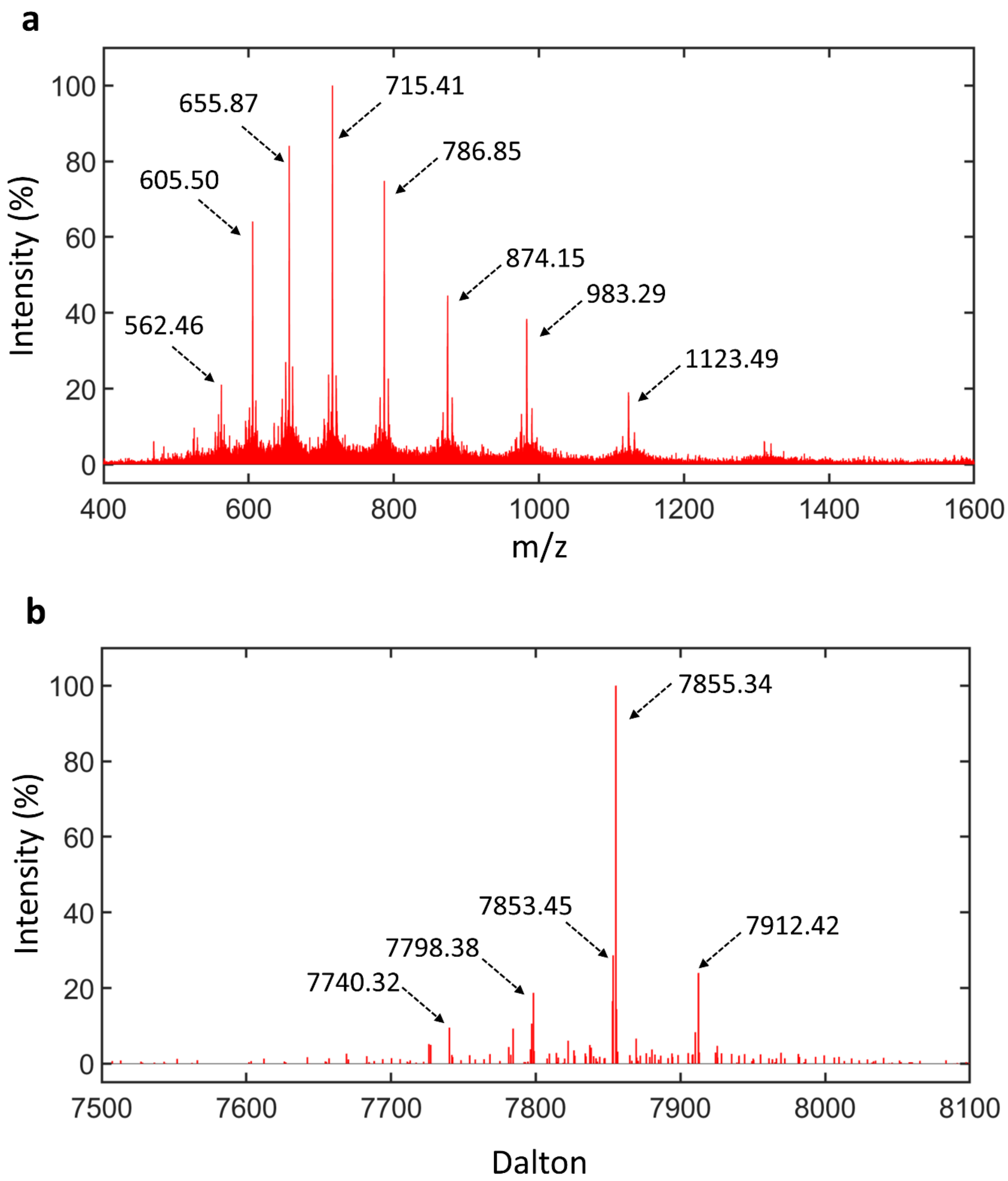

Supplementary Figure 14. Mass analysis of tick-GRP77 synthesized using Boc-based SPPS. (**a**) ESI-MS spectrum of tick-GRP77. (**b**) The observed deconvoluted mass (7855.34 Da, $[M+H]^+$) corresponds with the calculated monoisotopic mass (7853.74 Da). The deconvoluted mass corresponding to 7798.38 Da indicates a glycine residue deletion (- 57 Da) and the deconvoluted mass at 7740.32 Da is an aspartic acid residue deletion (- 115 Da). The 7912.42 Da mass could indicate a metal ion (+ 57 Da) chelated to the polypeptide.

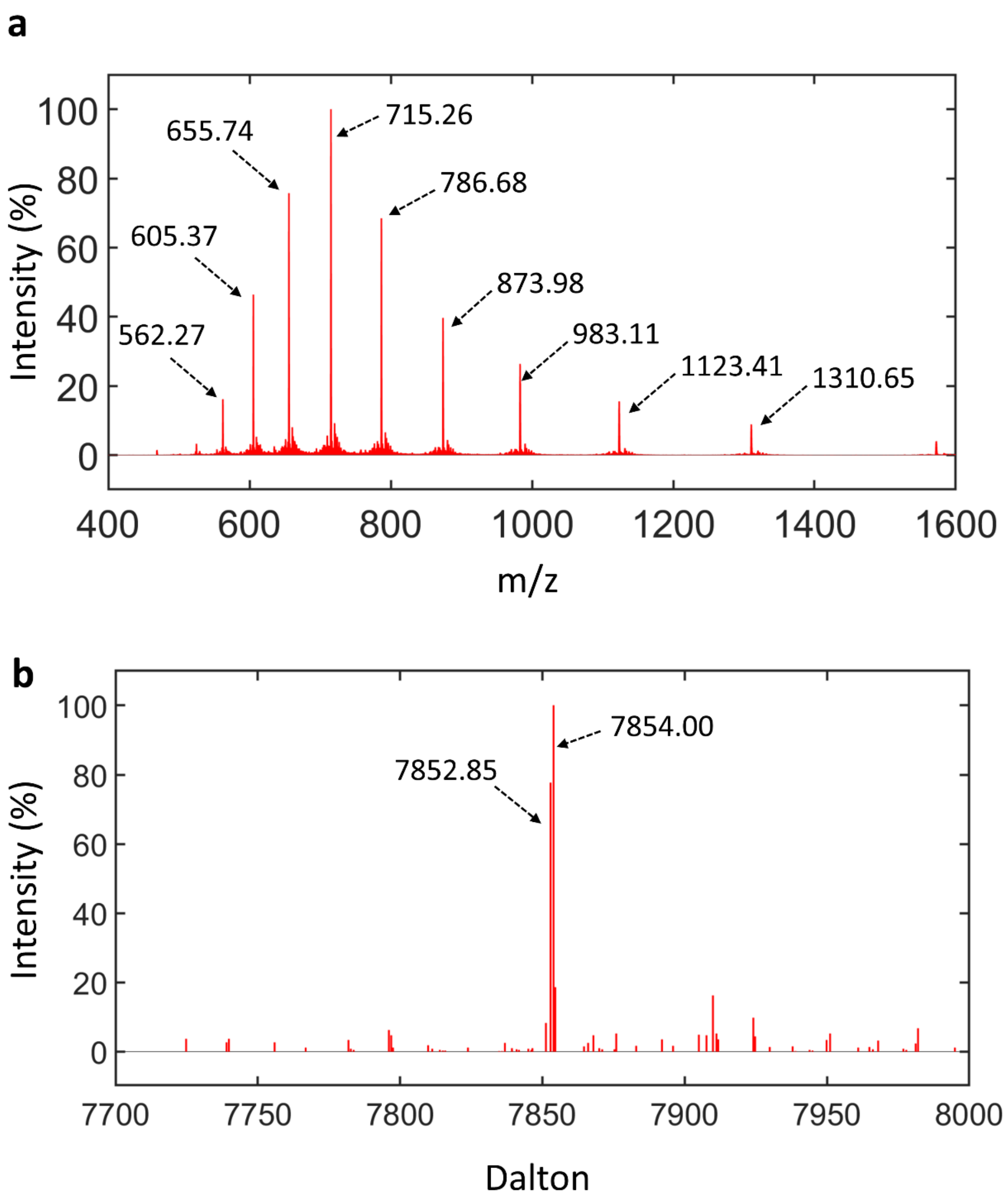

Supplementary Figure 15. Mass analysis of tick-GRP77 synthesized using Fmoc-based SPPS. (**a**) ESI-MS spectrum of tick-GRP77. (**b**) The deconvoluted mass (7854.00 Da, $[M+H]^+$) corresponds with the calculated monoisotopic mass (7853.74 Da) of tick-GRP77.

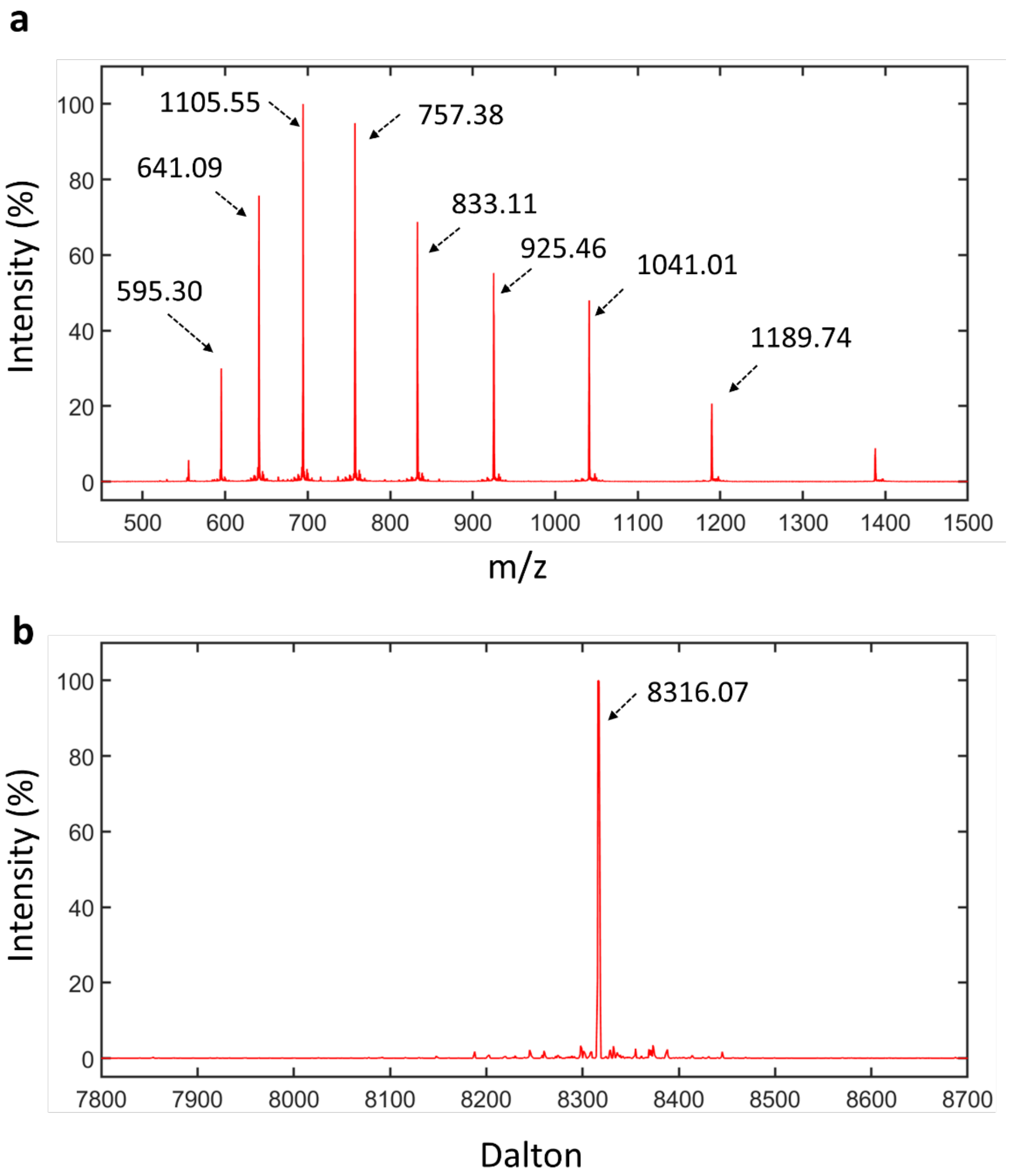

Supplementary Figure 16. Mass analysis of OG488-GRP77 synthesized using Boc-based SPPS. (**a**) ESI-MS spectrum of OG488-GRP77. (**b**) The deconvoluted mass (8316.07 Da, $[M+H]^+$) corresponds with the calculated monoisotopic mass (8317.07 Da) of OG488-GRP77.

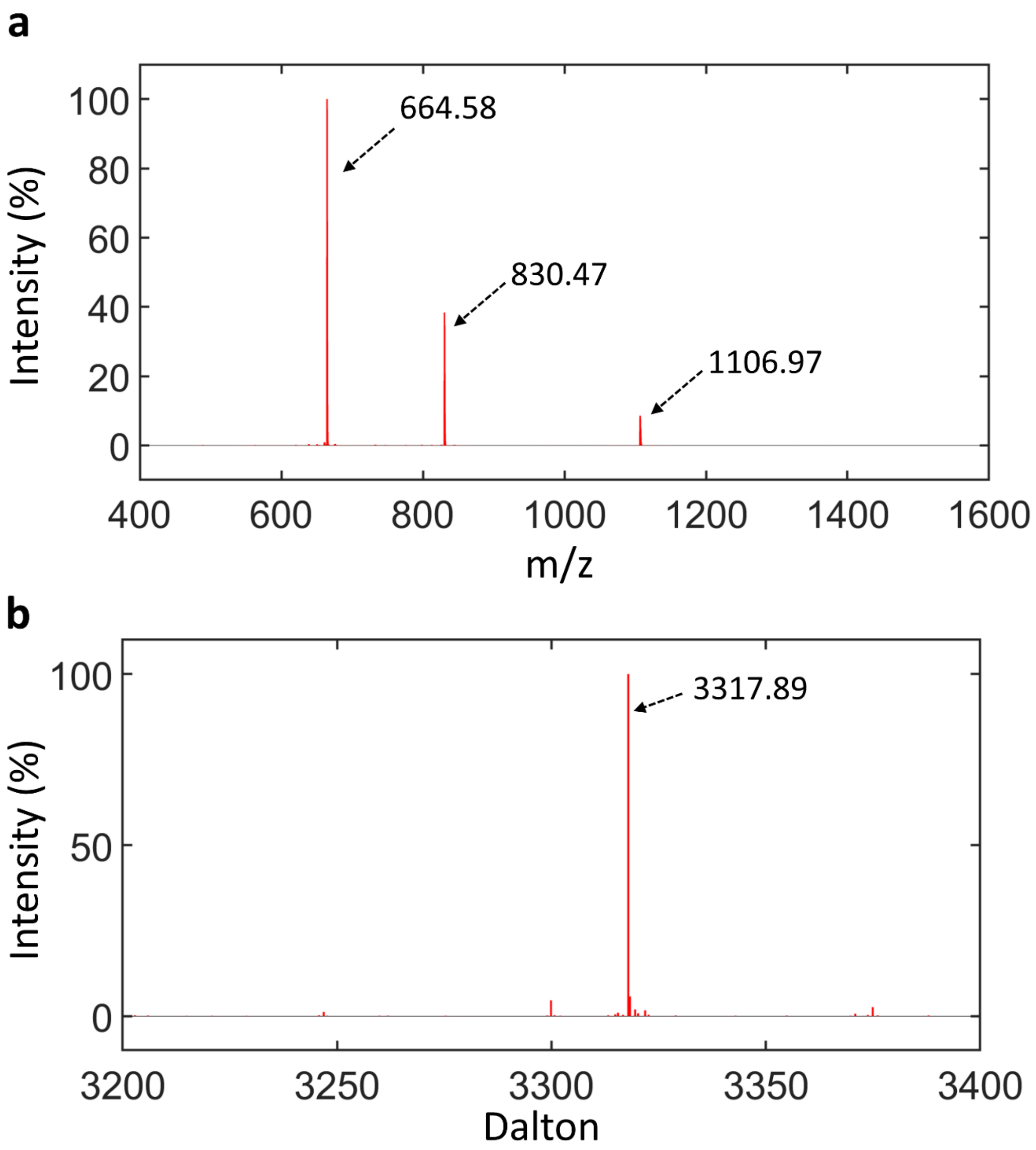

Supplementary Figure 17. Mass analysis of the N-terminus of tick-GRP77. (**a**) ESI-MS spectrum of N-terminus of tick-GRP77 synthesized using Boc-based SPPS. (**b**) The deconvoluted mass (3317.89 Da, $[M+H]^+$) corresponds with the calculated monoisotopic mass (3316.87) of the N-terminus.

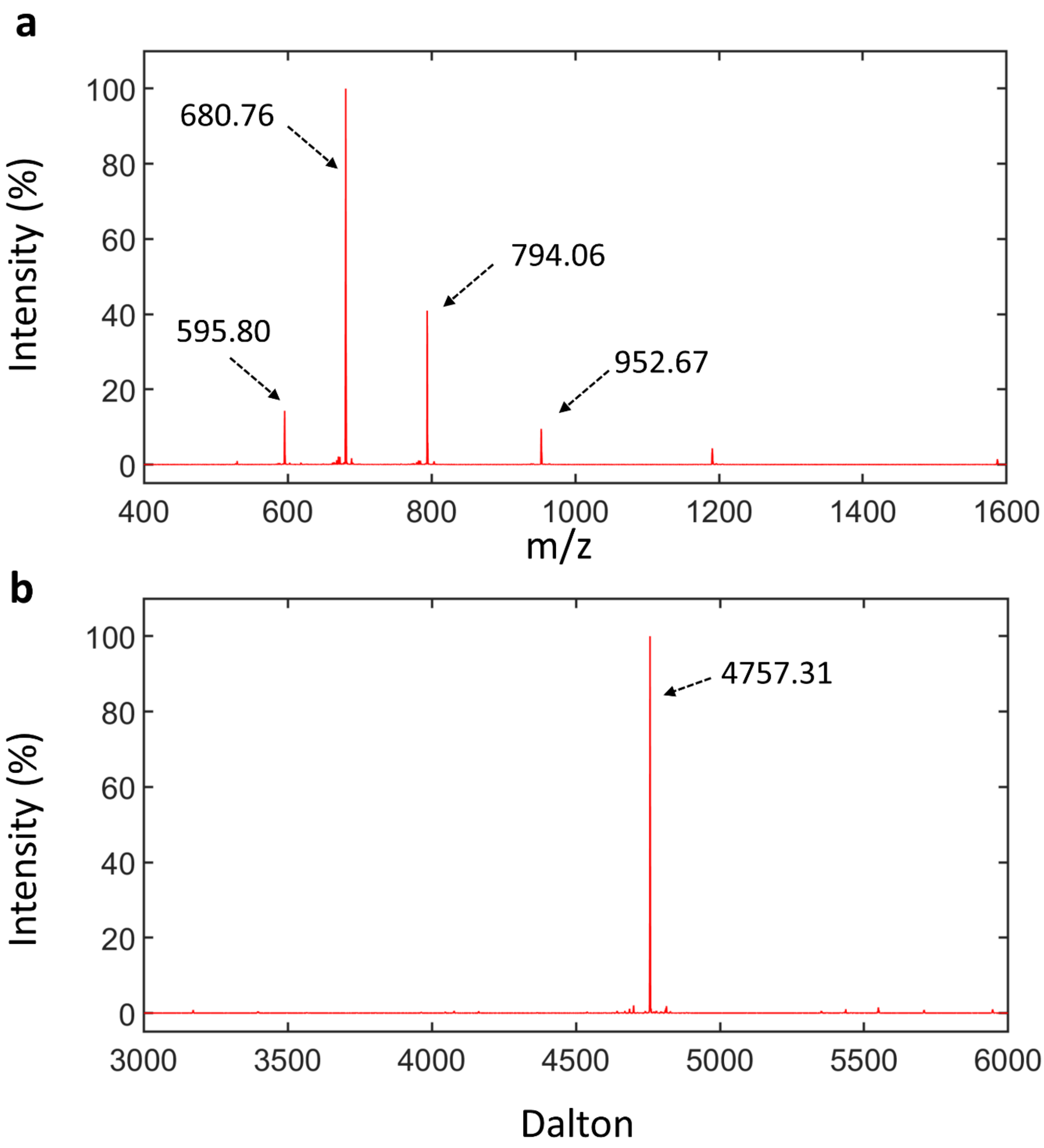

Supplementary Figure 18. Mass analysis of the C-terminus of tick-GRP77. (**a**) ESI-MS spectrum of C-terminus of tick-GRP77 synthesized using Fmoc-based SPPS. (**b**) The deconvoluted mass (4757.31 Da, $[M+H]^+$) corresponds with the calculated monoisotopic mass (4755.86) of the C-terminus.

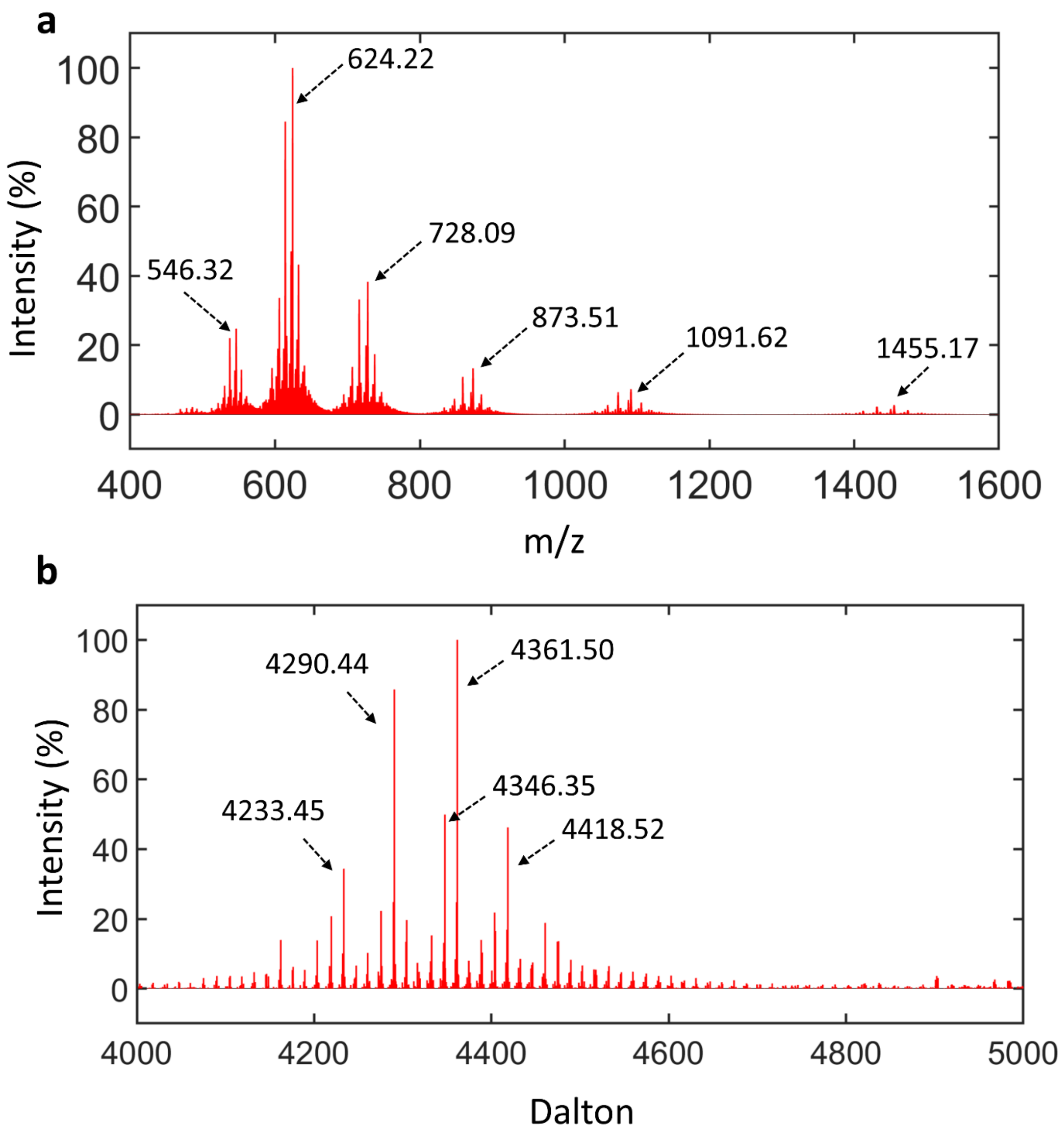

Supplementary Figure 19. Mass analysis of the ΔFY mutated C-terminus. (**a**) ESI-MS spectrum of the ΔFY mutant. (**b**) Deconvoluted and calculated monoisotopic mass of $[M+H]^+$ for the ΔFY mutant were 4361.50 Da and 4361.11 Da respectively. 4290.44 Da corresponds to an alanine residue deletion in the ΔFY mutant. 4233.45 Da corresponds to a glutamine residue deletion in the ΔFY mutant.

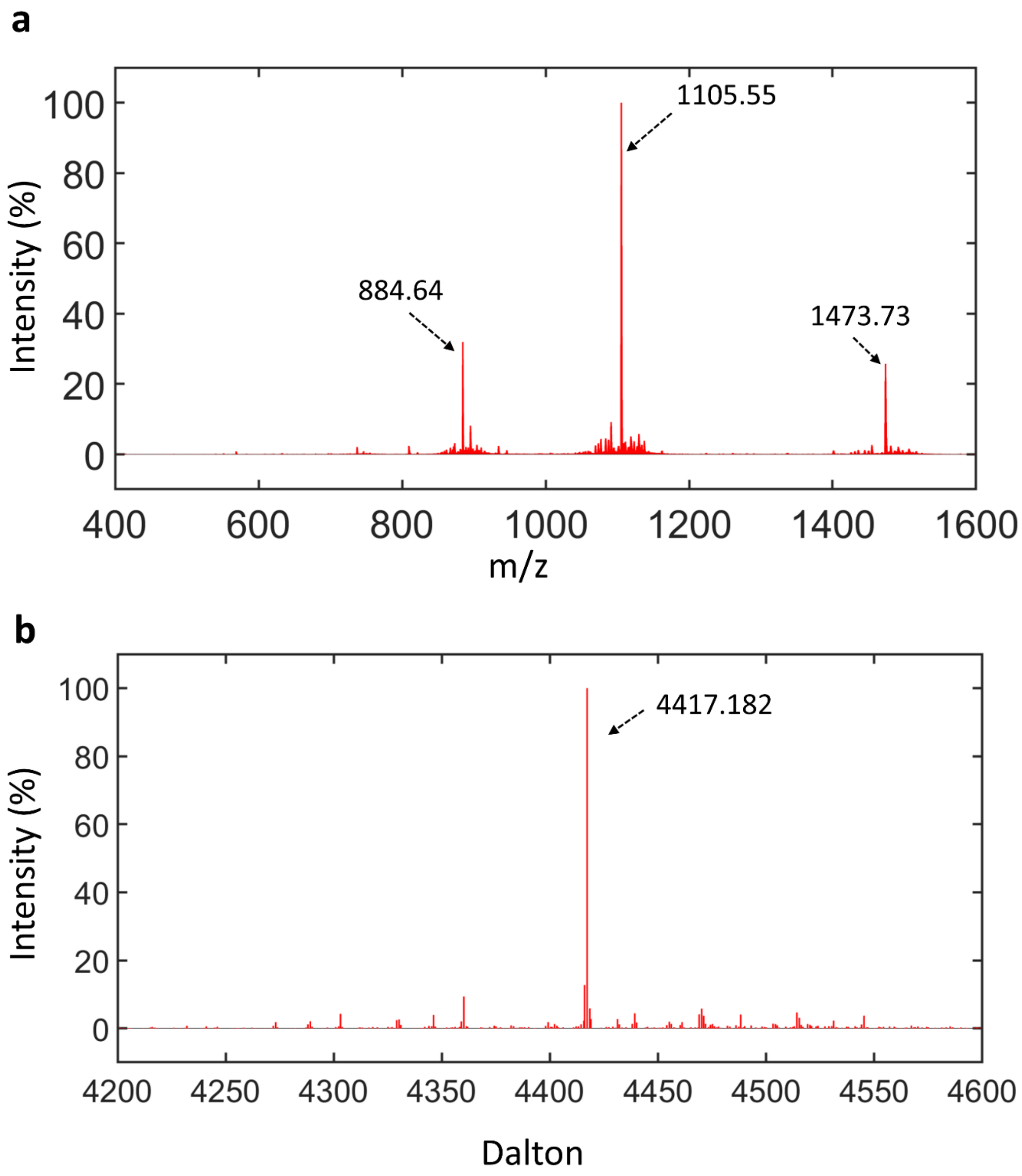

Supplementary Figure 20. Mass analysis of the ΔR mutated C-terminus. (**a**) ESI-MS spectrum of the ΔR mutant. (**b**) Deconvoluted and calculated monoisotopic mass of $[M+H]^+$ for the ΔR mutant were 4417.18 Da and 4417.01 Da respectively.

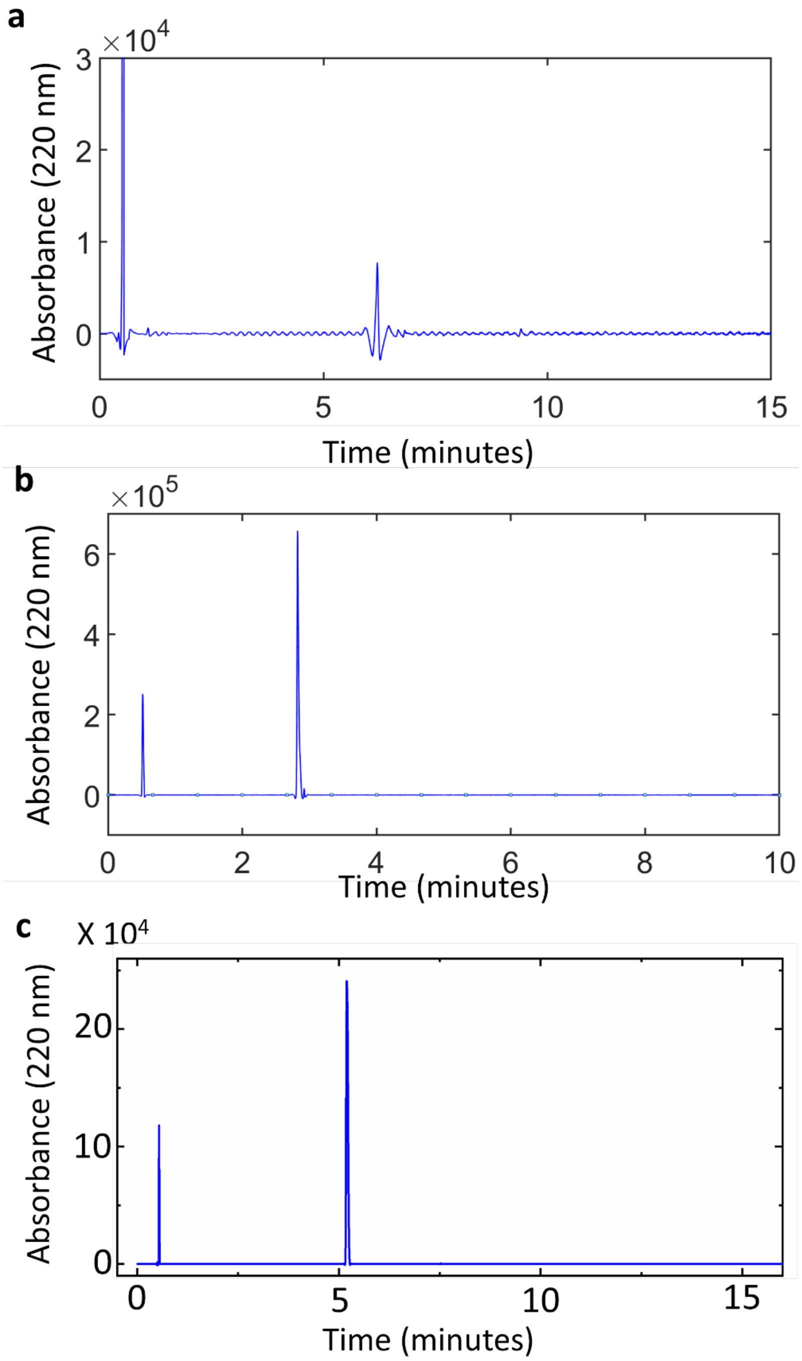

Supplementary Figure 21. HPLC chromatograms of tick-GRP77. (**a**) HPLC trace of the purified tick-GRP77 synthesized using Boc-based SPPS (220 nm). The retention time of the protein is approximately 6 minutes. (**b**) HPLC trace of the purified tick-GRP77 synthesized using Fmoc-based SPPS (220 nm). The retention time of the protein is approximately 3 minutes. The difference in retention time is due to the fact that different gradients have been used. The peak at 0.5 min is the injection peak. (**c**) HPLC trace of the purified OG488-GRP77 synthesized using Boc-based SPPS (220 nm). The injection peak is visible at 0.5 min and retention time of the protein is approximately 5 minutes.

a

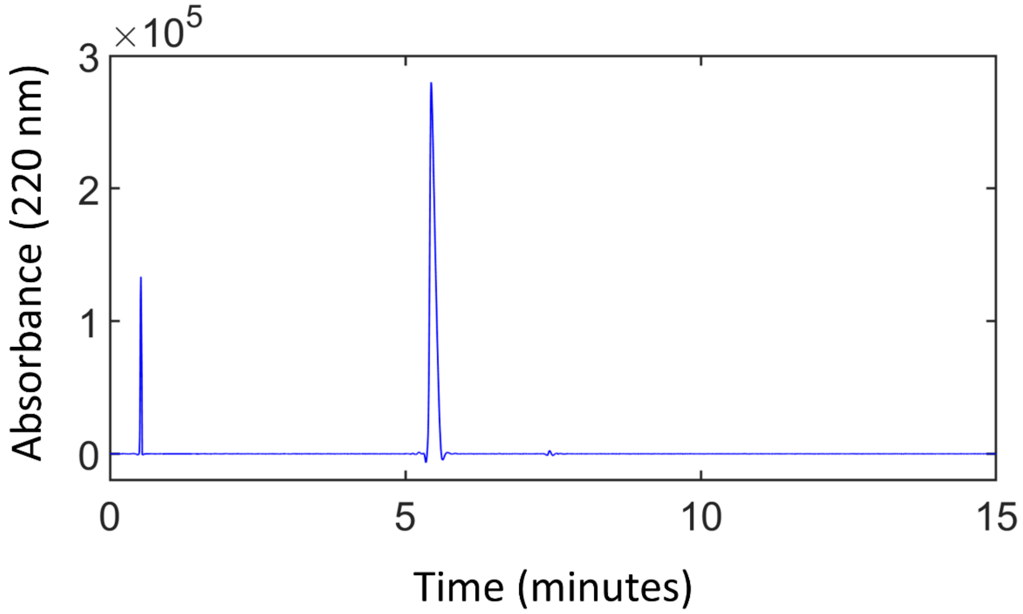

b

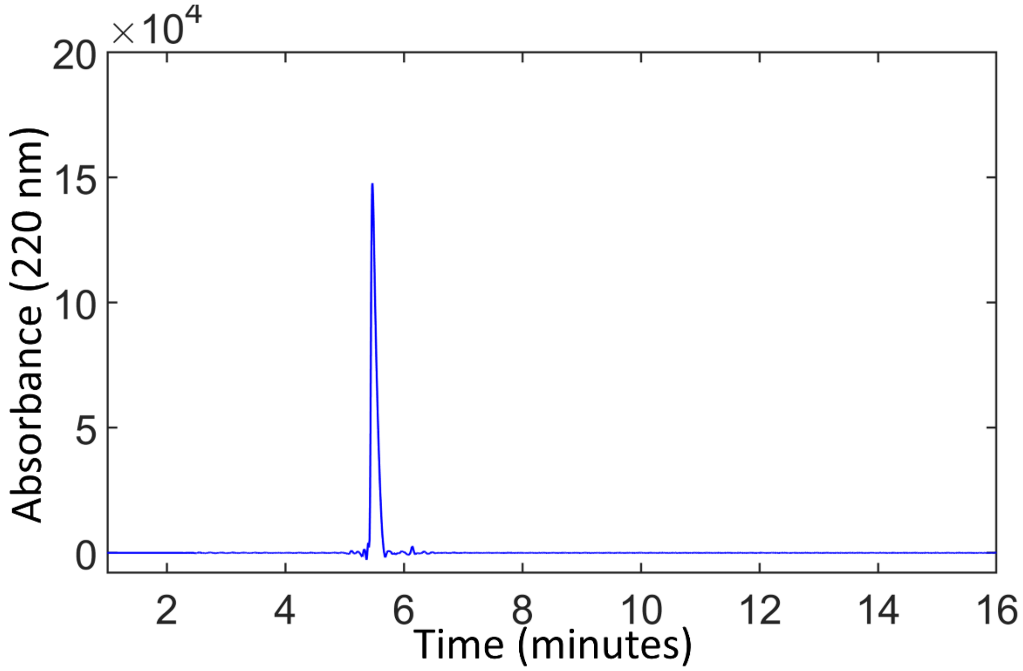

Supplementary Figure 22. HPLC chromatograms of the N- and C-terminus of tick-GRP77. (**a**) HPLC analysis of the purified N-terminus synthesized using Boc-based SPPS (220 nm). The peak at 0.5 min is the injection peak. (**b**) HPLC analysis of the C-terminus synthesized using Fmoc-based SPPS (220 nm).

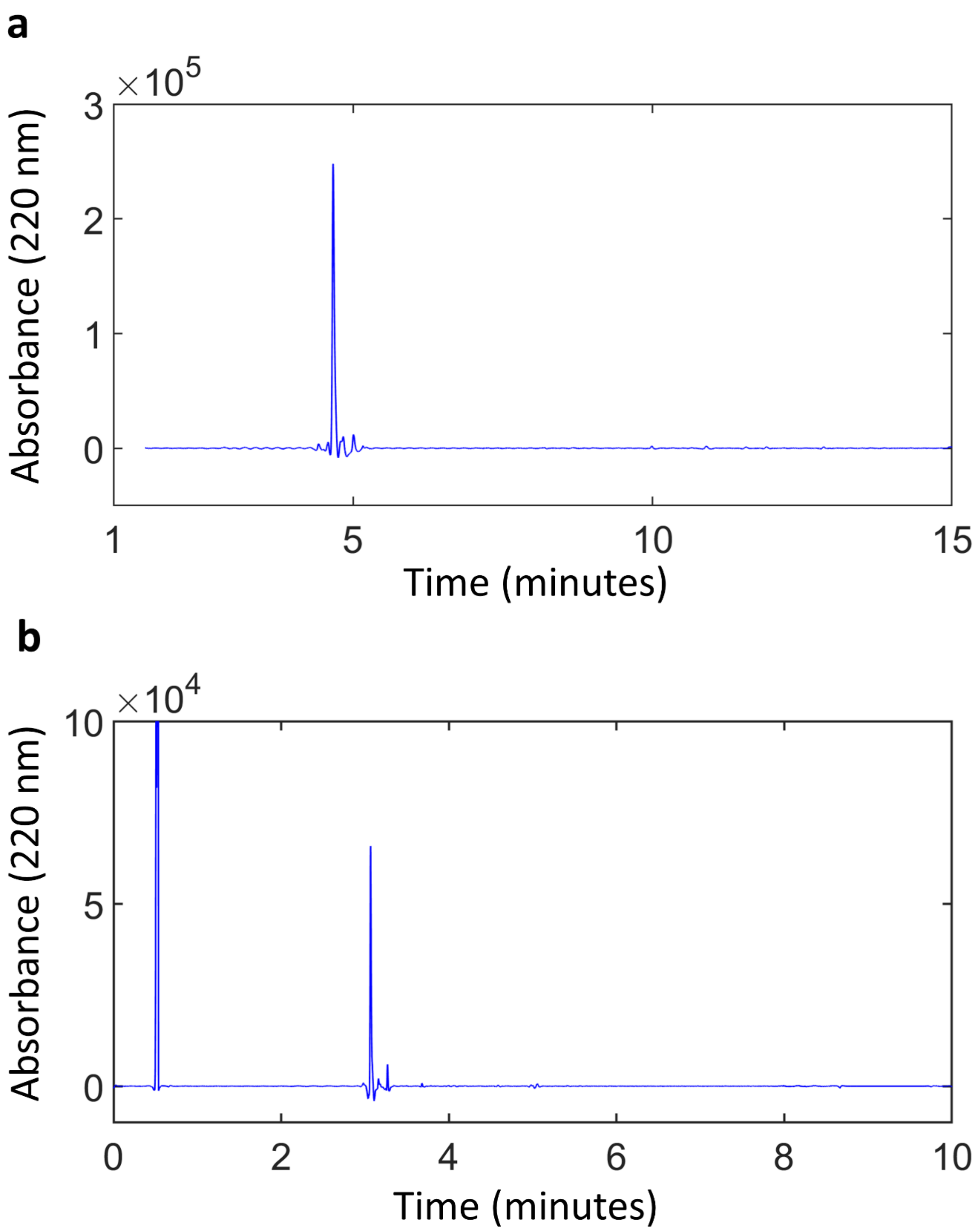

Supplementary Figure 23. HPLC chromatograms of the two C-terminus mutants. (**a**) HPLC analysis of the purified C- terminus ΔFY mutant synthesized using Boc-based SPPS (220 nm). (**b**) HPLC analysis of C-terminus ΔR mutant synthesized using Fmoc-based SPPS (220 nm). The peak at 0.5 min is the injection peak.

Section 3: References

1. Nuijens, T. et al. Improved solid phase synthesis of peptide carboxyamidomethyl (Cam) esters for enzymatic segment condensation. *Tetrahedron Lett.* **57**, 3635–3638 (2016).

2. Toplak, A. et al. From thiol-subtilisin to omniligase: Design and structure of a broadly applicable peptide ligase. *Comput. Struct. Biotechnol. J.* **19**, 1277–1287 (2021).

3. Jones, D. T. & Cozzetto, D. DISOPRED3: precise disordered region predictions with annotated protein-binding activity. *Bioinformatics* **31**, 857–863 (2015).

4. Ishida, T. & Kinoshita, K. PrDOS: prediction of disordered protein regions from amino acid sequence. *Nucleic Acids Res.* **35**, W460–W464 (2007).

5. Walsh, I., Martin, A. J. M., Di Domenico, T. & Tosatto, S. C. E. ESpritz: accurate and fast prediction of protein disorder. *Bioinformatics* **28**, 503–509 (2012).

6. Linding, R. et al. Protein disorder prediction: implications for structural proteomics. *Structure* **11**, 1453–1459 (2003).

7. Dass, R., Mulder F. A. A. & Nielsen, J. T. ODiNPred: comprehensive prediction of protein order and disorder. *Sci. Rep.* **10**, 14780 (2020).
