# Supplementary material for: Phase separation and ageing of glycine-rich protein from tick adhesive
Source: Nat Chem. 2024 Nov 29;17(2):186–97. doi: 10.1038/s41557-024-01686-8 (PMC11794139; doi:10.1038/s41557-024-01686-8)
Supplement: Supplementary file 2 — Reporting Summary [file 41557_2024_1686_MOESM2_ESM.pdf]

nature portfolio

Corresponding author(s): Siddharth Deshpande, Ingrid Dijkgraaf

Last updated by author(s): Aug 15, 2024

Reporting Summary

Nature Portfolio wishes to improve the reproducibility of the work that we publish. This form provides structure for consistency and transparency in reporting. For further information on Nature Portfolio policies, see our Editorial Policies and the Editorial Policy Checklist.

Statistics

For all statistical analyses, confirm that the following items are present in the figure legend, table legend, main text, or Methods section.

n/a	Confirmed	
☐	☒	The exact sample size (*n*) for each experimental group/condition, given as a discrete number and unit of measurement
☐	☒	A statement on whether measurements were taken from distinct samples or whether the same sample was measured repeatedly
☒	☐	The statistical test(s) used AND whether they are one- or two-sided<br>*Only common tests should be described solely by name; describe more complex techniques in the Methods section.*
☒	☐	A description of all covariates tested
☒	☐	A description of any assumptions or corrections, such as tests of normality and adjustment for multiple comparisons
☐	☒	A full description of the statistical parameters including central tendency (e.g. means) or other basic estimates (e.g. regression coefficient) AND variation (e.g. standard deviation) or associated estimates of uncertainty (e.g. confidence intervals)
☒	☐	For null hypothesis testing, the test statistic (e.g. *F*, *t*, *r*) with confidence intervals, effect sizes, degrees of freedom and *P* value noted<br>*Give P values as exact values whenever suitable.*
☒	☐	For Bayesian analysis, information on the choice of priors and Markov chain Monte Carlo settings
☒	☐	For hierarchical and complex designs, identification of the appropriate level for tests and full reporting of outcomes
☒	☐	Estimates of effect sizes (e.g. Cohen's *d*, Pearson's *r*), indicating how they were calculated

Our web collection on statistics for biologists contains articles on many of the points above.

Software and code

Policy information about availability of computer code

Data collection	Data was collected using Nikon-Ti2-Eclipse (NIS elements, version AR 5.41.00), Nikon C2 (NIS elements, version AR 5.41.00), Leica SP8-SMD (LAS-X, version 3.5.7.23225), and JPK ForceRobot 300 (JPK SPM Data Processing, version 6.0).
Data analysis	Data was analyzed using FIJI (Image J, version 1.5.2), MATLAB (version R2022b), Origin (version 2024), IUPred 2A (version 2A), CIDER (version 1.7), ODiNPred (version 1.0), ESpritz (version 1.3), DISOPRED 3 (version 3.16), PrDOS (version 1.0), and DisEMBL (version 1.5).

For manuscripts utilizing custom algorithms or software that are central to the research but not yet described in published literature, software must be made available to editors and reviewers. We strongly encourage code deposition in a community repository (e.g. GitHub). See the Nature Portfolio guidelines for submitting code & software for further information.

Data

Policy information about availability of data

All manuscripts must include a data availability statement. This statement should provide the following information, where applicable:

- Accession codes, unique identifiers, or web links for publicly available datasets
- A description of any restrictions on data availability
- For clinical datasets or third party data, please ensure that the statement adheres to our policy

Data supporting the findings of this study are available within the paper, Supplementary Information, Source Data and on figshare (https://doi.org/10.6084/m9.figshare.26490541).

Human research participants

Policy information about studies involving human research participants and Sex and Gender in Research.

Reporting on sex and gender	N/A
Population characteristics	N/A
Recruitment	N/A
Ethics oversight	N/A

Note that full information on the approval of the study protocol must also be provided in the manuscript.

Field-specific reporting

Please select the one below that is the best fit for your research. If you are not sure, read the appropriate sections before making your selection.

☒ Life sciences ☐ Behavioural & social sciences ☐ Ecological, evolutionary & environmental sciences

For a reference copy of the document with all sections, see nature.com/documents/nr-reporting-summary-flat.pdf

Life sciences study design

All studies must disclose on these points even when the disclosure is negative.

Sample size	Sample size was chosen to be at least three in case of statistical analyses and for majority of experiments, providing a balance between experimental variation and practical considerations. Some observations were repeated twice (phase diagrams and microfluidics) due to the large number of experiments involved and limited material availability. In general, each of the microscopic observations were based on a large number of condensates (tens to hundreds in a typical field-of-view) present in the recorded images, making a sample size of two already sufficient to reach a conclusion.
Data exclusions	No data were excluded.
Replication	All experiments were replicated at least twice.
Randomization	Since we did not compare multiple experimental groups, this is not relevant.
Blinding	Since we did not compare multiple experimental groups, this is not relevant.

Reporting for specific materials, systems and methods

We require information from authors about some types of materials, experimental systems and methods used in many studies. Here, indicate whether each material, system or method listed is relevant to your study. If you are not sure if a list item applies to your research, read the appropriate section before selecting a response.

Materials & experimental systems

n/a	Involved in the study	
☒	☐	Antibodies
☒	☐	Eukaryotic cell lines
☒	☐	Palaeontology and archaeology
☐	☒	Animals and other organisms
☒	☐	Clinical data
☒	☐	Dual use research of concern

Methods

n/a	Involved in the study	
☒	☐	ChIP-seq
☒	☐	Flow cytometry
☒	☐	MRI-based neuroimaging

Animals and other research organisms

Policy information about studies involving animals; ARRIVE guidelines recommended for reporting animal research, and Sex and Gender in Research

Laboratory animals	The study did not involve laboratory animals.
Wild animals	Ticks belonging to the species Ixodes ricinus at different life stages (larva, nymph and adult) were opportunistically collected using the tick-dragging method. A dragging cloth was swept over vegetation, and any ticks that attached to the cloth were aspirated into a Falcon tube with either some vegetation or small piece of wet tissue inside (for humidity) to be transported back to the lab and stored in a fridge. Ticks were killed as part of the dissection process, and any additional unused ticks were killed (because of desiccation and/or lack of blood meal) after the study to prevent any potential risk of releasing ticks back into the environment which could bite humans.
Reporting on sex	Adult female ticks were used to isolate the salivary glands because of their bigger size making them easier to dissect. Also, their salivary glands are bigger, so contain more material to work with.
Field-collected samples	Field-collected ticks were used in the study and treated as described above.
Ethics oversight	No ethical approval was required.

Note that full information on the approval of the study protocol must also be provided in the manuscript.
